# Pharmacological inhibition of Ubiquitin-Specific Peptidase 10 (USP10) with spautin-1 attenuates adipogenesis through CCAAT/Enhancer-Binding Protein Beta (C/EBPβ) destabilization

**DOI:** 10.1186/s43556-025-00389-x

**Published:** 2025-12-19

**Authors:** Zolzaya Erdenebileg, Desy Damayanti Simamora, Joong-Kwon Park, Rosana Nogueira, Young Bin Kim, Jeong-Yun Choi, Hyeon-Gu Kang, Hack Sun Choi, Jung-Hwan Baek, Kyung-Hee Chun

**Affiliations:** 1 Department of Biochemistry & Molecular Biology, Yonsei University College of Medicine, 50-1 Yonsei-Ro, Seodaemun-Gu, Seoul, 03722 Republic of Korea; 2 School of Medical Science, Brain Korea 21 Project, Yonsei University College of Medicine, Seoul, Republic of Korea; 3 JLBiotherapeutics, Yongin-si, Republic of Korea; 4 Affiliate Faculty, Pohang University of Science and Technology, Pohang, Republic of Korea

**Keywords:** Spautin-1, Adipogenesis, Lipid accumulation, Ubiquitin‐specific peptidase, Deubiquitination, C/EBPβ

## Abstract

The pharmacological control of lipid accumulation in white adipose tissue (WAT) is a key area of focus in obesity research, yet the role of deubiquitination in adipocyte lipid storage remains underexplored. We found that spautin-1, an inhibitor of the deubiquitinases ubiquitin-specific peptidase 10 (USP10) and 13 (USP13), suppressed lipid accumulation during adipogenesis. Therefore, we investigated whether blocking deubiquitination restricts adipogenesis and acts as the underlying mechanism. Mining public datasets revealed that USP10 expression is substantially increased in the adipose tissue (AT) from individuals with obesity. Moreover, USP10 exhibited a depot-specific expression pattern, with higher levels in visceral AT than in subcutaneous AT, whereas no such difference was observed for USP13. Consistently, in high-fat diet–fed mice, USP10 was markedly upregulated in gonadal WAT, whereas USP13 was undetectable. Genetic ablation of USP10 phenocopied spautin-1 treatment by reducing the expression of the components of the peroxisome proliferator-activated receptor gamma (PPARγ)/CCAAT/enhancer-binding protein alpha (C/EBPα) axis, while USP13 knockdown induced minimal effects, thus implicating USP10 as the principal mediator. Mechanistically, USP10 directly interacted with C/EBPβ and stabilized it via deubiquitination. However, spautin-1 or USP10 knockdown enhanced C/EBPβ ubiquitination and proteolysis, thereby impairing the adipogenic commitment. The overexpression of wild-type USP10, but not its catalytically inactive mutant, rescued C/EBPβ stability, thus confirming the requirement for its enzymatic activity. The administration of spautin-1 to high-fat diet–fed mice mitigated body weight gain and reduced adipose tissue mass in vivo. Notably, spautin-1 selectively suppressed USP10 and C/EBPβ in gonadal WAT without affecting the liver, which highlights the tissue-specific pharmacodynamics. Collectively, these findings define the USP10–C/EBPβ axis as a key regulator of adipogenesis and position spautin-1 as a mechanistically grounded anti-obesity candidate that warrants translational evaluation.

**Supplementary Information:**

The online version contains supplementary material available at 10.1186/s43556-025-00389-x.

## Introduction

Obesity is a primary risk factor of metabolic diseases, including type 2 diabetes (T2D), cardiovascular disease, and metabolism dysfunction-associated steatohepatitis (MASH). The prevalence of obesity has increased substantially over the past two decades and poses a major burden on public health worldwide [1]. The World Health Organization defines obesity as a condition of abnormal or excessive fat accumulation that presents a health risk, and it affects more than 40% of adults worldwide [1, 2]. This fat storage is regulated by cellular processes called hyperplasia and hypertrophy in cells. Adipogenesis is a hyperplastic process that generates new adipocytes from preadipocyte precursors, which undergo considerable hypertrophic expansion via intracellular lipid accumulation [3, 4]. Under normal conditions, adipogenesis is beneficial for maintaining insulin sensitivity and providing safe lipid storage. However, the balance between hypertrophy and adipogenesis influences metabolic health, with larger and smaller adipocytes associated with insulin resistance and improved metabolic outcomes. As adipocytes enlarge, they exhibit elevated lipolysis and increasingly release proinflammatory cytokines and reduce the secretion of anti-inflammatory adipokines [3]. Therefore, inhibiting adipogenesis might be a valid therapeutic strategy to reduce inflammation and prevent further disease progression. Although several anti-obesity drugs have been approved by the United States Food and Drug Administration (FDA), their overall efficacy remains limited, thus highlighting an unmet clinical need. For instance, glucagon-like peptide-1 receptor agonists (GLP-1 RAs) have transformed the management of metabolic disease by reducing body weight. However, their broader adoption is hindered by frequent gastrointestinal adverse events, attenuation of efficacy over time, high costs, injectable formulations, and uncertainties about long-term safety and sustainability of benefits [5–7]. The clinical impact and commercial success of GLP-1 RAs have demonstrated the medical feasibility and substantial market potential of pharmacological obesity therapies, catalyzing renewed interest in alternative molecular targets and modalities. These considerations underscore the urgency of diversifying therapeutic mechanisms that can enhance metabolic regulation and support sustained weight loss.

Ubiquitin-specific proteases (USPs) are a major subclass of deubiquitinases (DUBs) that regulate key signaling pathways, including DNA damage responses, cellular tumor antigen p53 (p53) regulation, and transforming growth factor beta (TGF-β) signaling, thereby positioning them as attractive drug targets [8]. Emerging evidence has implicated USPs in metabolic control, where they modulate disease processes by removing ubiquitin chains from specific substrates. For instance, USP22 promotes lipidome accumulation and PPARγ deubiquitination in hepatocellular carcinoma cells [9], while the inhibition or deletion of other DUBs, such as USP1 and USP15, attenuates adipogenic processes and steatotic liver phenotypes [10]. These observations motivate the development of DUB-targeted therapeutics with improved selectivity and translational potential.

Spautin-1 is a small-molecule autophagy inhibitor that suppresses the deubiquitinating activity of USP10 and USP13 and promotes the degradation of phosphatidylinositol 3-kinase 34 (Vps34) phosphatidylinositol 3-kinase (PI3-kinase) [11]. Moreover, spautin-1 treatment reduces lipopolysaccharide-induced inflammation by inhibiting USP10 and stabilizing the nuclear factor (NF)-κB essential modulator (NEMO), which links the inhibitor of NF-κB kinase subunit alpha and beta (IKKα and IKKβ) during the macrophage inflammation responses [12]. Furthermore, spautin-1 injection promotes myeloma cell apoptosis by inhibiting USP10, which is associated with the degradation of cyclin D3 [13]. When spautin-1 was initially explored in oncology, it exhibited activity across tumor contexts by limiting the USP10-dependent pathways and, in some settings, sensitizing cells via USP13 inhibition [14–19]. Beyond cancer applications, spautin-1 has demonstrated benefits in neurological and ischemic models [20, 21]. Despite these advances, whether spautin-1 can limit adipogenesis and lipid accumulation and, critically, whether these effects occur specifically via USP10 and/or USP13 in adipose tissue remains insufficiently elucidated.

In this study, we aimed to determine whether the pharmacological inhibition of deubiquitination controls adipogenesis and lipid accumulation and to define which DUB–substrate axis mediates these effects in adipose tissue. We hypothesized that spautin-1 primarily attenuates the lipid-storage processes through the reduced stability of DUB-sensitive factors that govern lipogenesis to limit hypertrophy and inflammatory output, while not deliberately targeting the early lineage commitment. To test this hypothesis, we integrated the mining of public adipose-tissue datasets with in vitro adipocyte differentiation assays, pharmacologic perturbation, and the genetic manipulation of USP10/USP13 and evaluated the anti-obesity efficacy in a high-fat diet mouse model. In brief, our results indicated that blocking deubiquitination reduces adipogenesis and neutral-lipid deposition; the genetic perturbations of USP10 and USP13 delineate their relative contributions to these processes; and spautin-1 mitigates weight gain and inhibits adipose-tissue expansion with depot-selective pharmacodynamics in vivo.

## Results

### Spautin-1 inhibits lipid accumulation and adipogenic/lipogenic programs during 3T3-L1 adipocyte differentiation

**Fig. 1:**
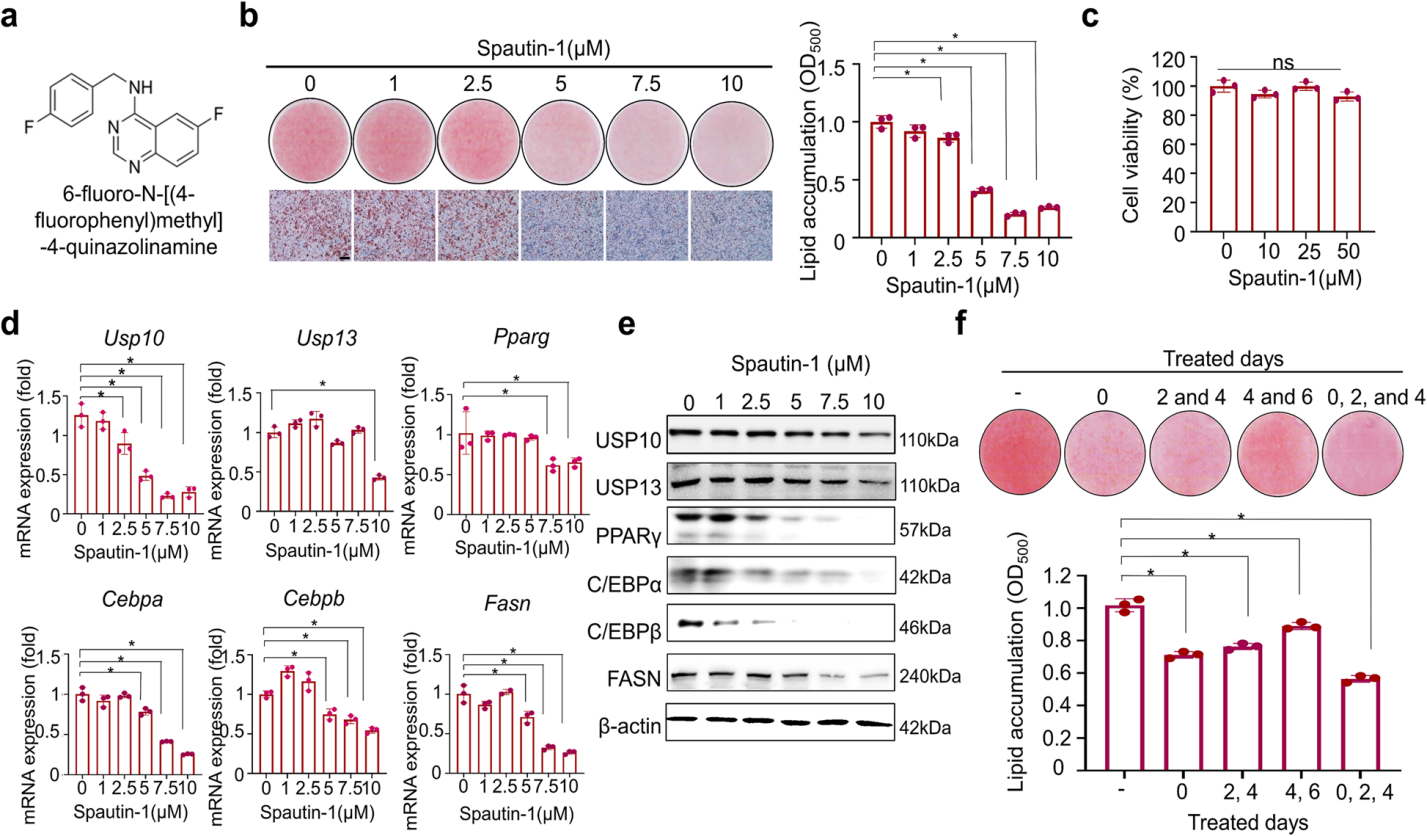
Spautin-1 attenuates adipogenesis and lipid accumulation in 3T3-L1 cells. All phenotypes were observed in 3T3-L1 cells. **a** Chemical structure of spautin-1. **b** Representative ORO images and quantification on Day 6 of MDI-induced differentiation. Cells were treated with spautin-1 at concentrations of 0, 1, 2.5, 5, 7.5, or 10 μM (vehicle = 0.1% DMSO), which were administered twice (Days 2 and 4). Bound dye was eluted in 100% isopropanol and measured at OD₅₀₀; values were normalized to those of the vehicle. Scale bar = 100 μm. **c** Cell viability after 72 h of exposure to spautin-1 and assessed by EZ-Cytox (OD₄₅₀); data were normalized to those of the vehicle (= 100%). **d** RT-qPCR graphs for DUBs (*Usp10* and *Usp13*) and adipogenic/lipogenic genes (*Pparγ, Cebpa, Cebpb,* and *Fasn*) on Day 6 of differentiation to mature adipocytes across the different spautin-1 doses; β-actin served as the reference gene. Data is shown as the mean fold-change relative to that of the vehicle. **e** Immunoblot images for the same proteins (USP10, USP13, PPARγ, C/EBPα, C/EBPβ, and FASN); β-actin served as the loading control. **f** Representative ORO images and quantification on Day 6 of MDI-induced differentiation. 3T3-L1 cells were differentiated into mature adipocytes and treated with spautin-1 either on Day 0 once, on Days 2 and 4, on Days 4 and 6, or on Days 0, 2, and 4. Bound dye was eluted in 100% isopropanol and measured at OD₅₀₀; values were normalized to those of the vehicle. Data were presented as the mean ± SD from *n *= 3 independent experiments (RT-qPCR was performed in technical triplicates). Statistical analyses: one-way ANOVA with Dunnett’s post hoc vs. vehicle for multi-dose comparisons; where applicable, two-tailed unpaired *t*-test. Significance: *p < 0.05. USP, ubiquitin-specific peptidase; ORO, Oil Red O; MDI, IBMX, dexamethasone, and insulin; DMSO, dimethyl sulfoxide; DUB, deubiquitinase; RT-qPCR, real-time quantitative polymerase chain reaction; SD, standard deviation; ANOVA, analysis of variance

To determine whether spautin-1 inhibits lipid accumulation during 3T3-L1 adipocyte differentiation and regulates the adipogenic/lipogenic programs, we established a differentiation protocol based on a combination of IBMX, dexamethasone, and insulin (MDI) (Fig. S1a–b). Successful induction was verified through time-dependent increases in PPARγ, C/EBPβ, C/EBPα, fatty acid synthase (FASN), and fatty acid-binding protein 4 (FABP4) at the mRNA and protein levels, normalized to β-actin (Fig. S1c–d). The chemical structure of spautin-1 is shown in Fig. 1a. The cells were then treated with either the vehicle (dimethyl sulfoxide (DMSO)) or five concentrations of spautin-1, which were administered twice at 2-day intervals after MDI induction. Neutral lipid accumulation was quantified on Day 6 by Oil Red O (ORO) staining (Fig. 1b). Spautin-1 produced a dose-dependent reduction in ORO-positive lipid droplets relative to those of the DMSO-treated controls (Fig. 1b), while cell viability remained unaffected across the working dose range (Fig. 1c).

Spautin-1 decreased the expression of the DUBs USP10 and USP13 together with key adipogenic/lipogenic regulators (PPARγ, C/EBPα, C/EBPβ, and FASN) at the transcript (quantitative polymerase chain reaction (qPCR)) and protein (immunoblot with densitometry) levels (Fig. 1d–e) in a dose-dependent manner. Moreover, when spautin-1 was applied after the initial 2-day mitotic clonal expansion (MCE) phase, specifically between Days 2–4 and 4–6 as shown in Fig. 1f (i.e., during the period in which differentiation is largely established and lipogenesis promotes lipid build-up), it still significantly suppressed lipid accumulation. In addition, we performed two key experiments demonstrating that spautin-1 effectively modulates autophagy markers. During adipogenic induction, the levels of beclin-1 (BECN1) protein and microtubule-associated protein 1 light chain 3 beta (MAP1LC3B) increased (Fig. S2a), indicating that autophagy was activated as the preadipocytes differentiated. Second, spautin-1 treatment significantly reduced the protein and mRNA levels of BECN1 and MAP1LC3B in 3T3-L1 cells (Fig. S2b–c), which was consistent with the pharmacological suppression of the autophagy program.

Collectively, these results indicate that spautin-1 suppresses lipid accumulation during 3T3-L1 adipocyte differentiation without detectable cytotoxicity and is accompanied by the coordinated downregulation of USP10/USP13 and adipogenic/lipogenic factors.

### USP10 is elevated in obesity and required for adipocyte differentiation

**Fig. 2:**
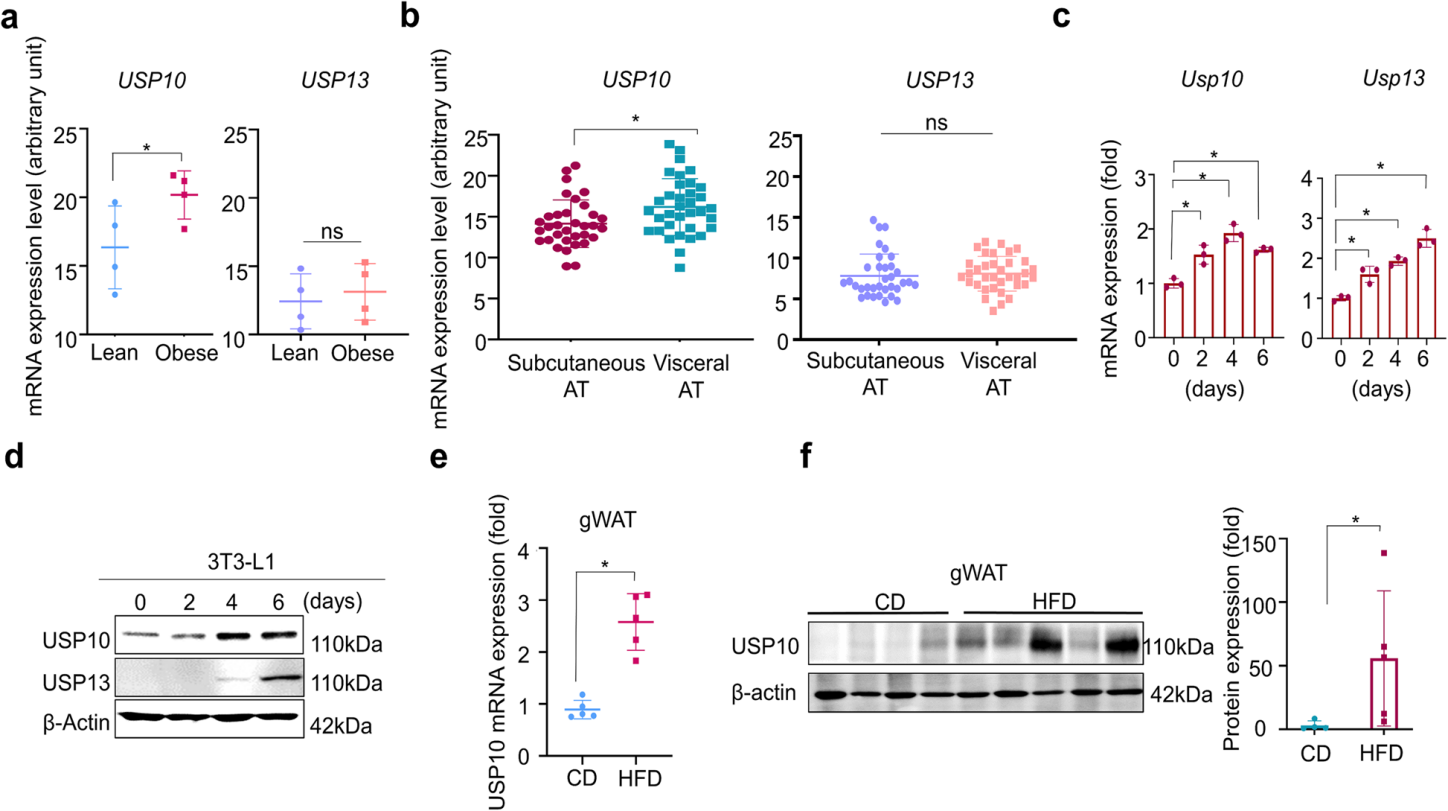
Expression of USP10 and USP13 in adipocytes and adipose tissue. **a** mRNA expression levels of *USP10* and *USP13* in the visceral adipose tissue of lean and obese adults (GSE235696). **b** mRNA expression levels of *USP10* and *USP13* in SAT and VAT (GSE213058). **c**–**d** mRNA and protein expression levels of USP10 and USP13 in 3T3-L1 cells during differentiation into mature adipocytes; β-actin served as the loading control. **e**–**f** USP10 mRNA and protein expression levels in gWAT of mice fed with CD or HFD. USP10 levels were normalized to β-actin and expressed relative to the control (= 1.0). Data were presented as the mean ± SD from three independent experiments (RT-qPCR was performed in technical triplicates). Statistical analyses: one-way ANOVA with Dunnett’s post hoc vs. the control, or a two-tailed unpaired *t-*test was used for specified pairwise comparisons. Significance: *p < 0.05. USP, ubiquitin-specific peptidase; SAT, subcutaneous adipose tissue; VAT, visceral adipose tissue; gWAT, gonadal white adipose tissue; CD, chow diet; HFD, high-fat diet; SD, standard deviation; RT-qPCR, real-time quantitative polymerase chain reaction; ANOVA, analysis of variance

As spautin-1 is a dual inhibitor of USP10 and USP13, we first assessed which target was most relevant in adipose tissue. Mining the publicly available clinical datasets, GTEx v8 (Adipose-Visceral [Omentum] and Adipose-Subcutaneous), and an obesity case–control adipose cohort (GEO accession placeholders: GSE235696, GSE213058), we observed that *USP10* was significantly upregulated in adipose tissue from individuals with obesity than in the lean individuals, whereas *USP13* exhibited no group-specific difference (Fig. 2a). Depot-wise analysis further revealed a depot-specific pattern for *USP10*, with higher expression in the visceral adipose tissue (VAT) than in the subcutaneous adipose tissue (SAT). In contrast, *USP13* expression did not differ between depots and generally exhibited a low abundance (Fig. 2b).

We next sought independent support through experimental models. During 3T3-L1 adipogenesis, we quantified the transcripts by qPCR and assessed protein abundance by immunoblotting. The mRNA and protein levels of USP10 and USP13 increased over time (Fig. 2c–d). However, USP10 increased earlier and more prominently, whereas USP13 increased primarily during the later stages in protein levels. The mRNA and protein levels of USP10 were markedly upregulated in the gonadal white adipose tissue (gWAT) of high-fat diet (HFD)–fed mice in vivo (Fig. 2e–f). However, whereas USP10 levels in inguinal WAT (iWAT) were below the limit of detection under our experimental conditions, USP13 was not detected in either depot.

**Fig. 3:**
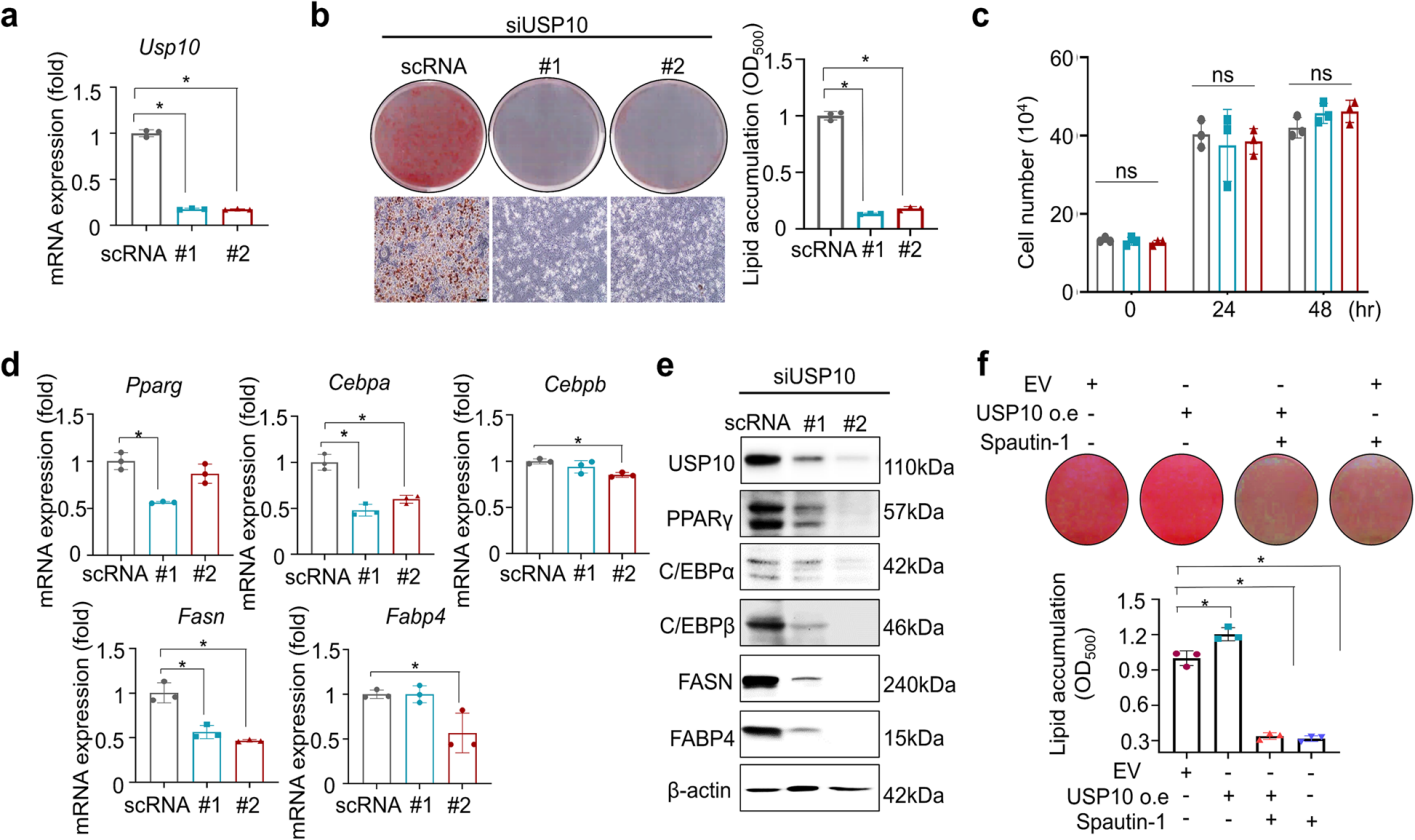
USP10 knockdown inhibits 3T3-L1 adipocyte differentiation and downregulates the adipogenic/lipogenic programs. All phenotypes were observed in 3T3-L1 cells. **a** Knockdown efficiency: USP10 mRNA levels by RT-qPCR at 48 h in cells transfected with siControl (scRNA) or two independent siUSP10 oligos (#1, #2; Supplementary Table S1), normalized to β-actin and expressed relative to the scRNA (= 1.0). **b** Adipogenesis readout: Representative ORO images on Day 6 and corresponding OD₅₀₀ quantification after dye elution; values were normalized to those of the scRNA (= 1.0). Scale bar = 100 μm. **c** MCE: Cell numbers at 48 h post-transfection were measured on a NanoEnTek automated counter with trypan blue exclusion. **d** RT-qPCR graphs for adipogenic/lipogenic genes (*Pparg*, *Cebpa*, *Cebpb*, *Fasn*, and *Fabp4*) treated with scRNA, siUSP10 #1, or siUSP10 #2; β-actin served as a reference gene; data are presented as mean ± SD (technical triplicates). **e** Immunoblot images for USP10 and adipogenic/lipogenic proteins (e.g., PPARγ, C/EBPα, C/EBPβ, FASN, and FABP4); β-actin served as the loading control. **f** 3T3-L1 preadipocytes were transduced to overexpress USP10, induced to differentiate at Day 0 with MDI, and then treated with or without spautin-1 (Days 0–4). On Day 6, the cells were fixed and stained with ORO. Representative ORO images on Day 6 and corresponding OD₅₀₀ quantification after dye elution; values were normalized to those of the control (EV only). Data are presented as the mean ± SD of *n* = 3 independent experiments. Statistics: one-way ANOVA with Dunnett’s post hoc vs. scRNA; for specified pairwise comparisons, a two-tailed unpaired *t*-test was used. Significance: *p < 0.05. USP, ubiquitin-specific peptidase; ORO, Oil Red O; MCE, mitotic clonal expansion; RT-qPCR, real-time quantitative polymerase chain reaction; ANOVA, analysis of variance; SD, standard deviation

To evaluate the functional contribution of each DUB, 3T3-L1 preadipocytes were transfected with small interfering RNAs (siRNAs) targeting USP10 or USP13 and induced to differentiate. ORO staining on Day 6 revealed that lipid accumulation was significantly reduced in the siUSP10-transfected cells compared with siControl (scRNA), whereas siUSP13 produced a noticeably smaller effect on the transfected cells (Fig. 3a–b; Fig. S3a–b). Neither of the two independent siRNAs against USP10 altered the cell counts at 24 and 48 h post-transfection (Fig. 3c), indicating that the reduced lipid accumulation was not attributable to cytotoxicity or impaired proliferation. Consistent with the phenotypic results, USP10 knockdown significantly decreased mRNA (Fig. 3d) and protein levels of PPARγ, C/EBPα, C/EBPβ, FABP4, and FASN (Fig. 3e). In 3T3-L1 cells, USP10 overexpression increased the lipid accumulation, and spautin-1 treatment effectively suppressed this increase (Fig. 3f). In contrast, siUSP13 produced only modest or negligible changes in these markers (Fig. S3c-d). Furthermore, USP10 knockdown led to a decrease in USP13 protein expression, whereas USP13 knockdown did not affect the USP10 levels (Fig. S3d).

Together, these results demonstrate that USP10 is a key DUB associated with adipogenesis and obesity, which serves as the functional target of spautin-1 in adipose tissue.

### USP10 interacts with and stabilizes C/EBPβ via direct deubiquitination

**Fig. 4:**
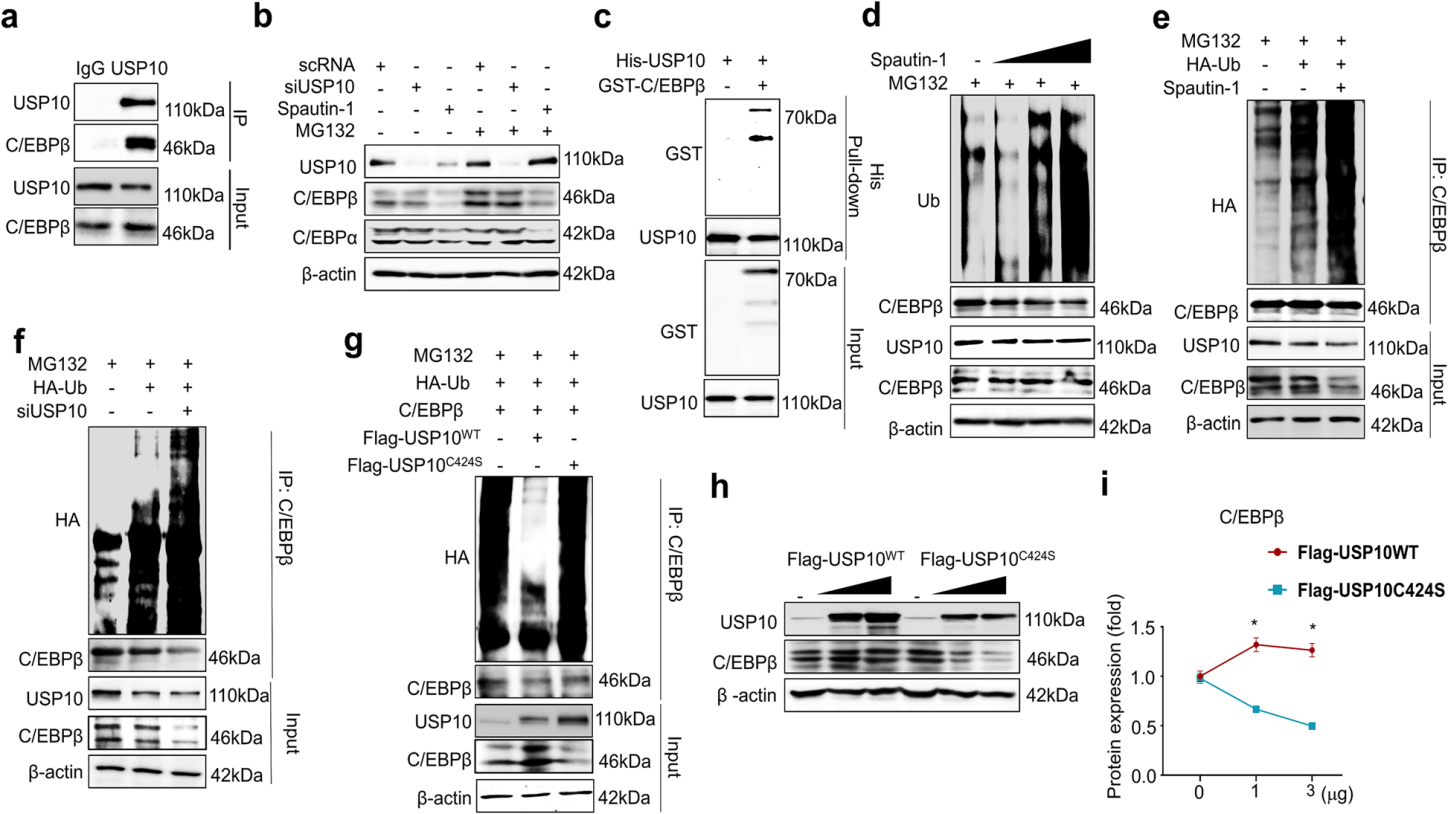
USP10 interacts with C/EBPβ and regulates its protein stability. All phenotypes were observed in 293FT cells. **a** Interaction between endogenous USP10 and C/EBPβ was confirmed. Cells were immunoprecipitated using IgG and USP10 antibodies. **b** Effect of USP10 knockdown and spautin-1 treatment on the protein stability of C/EBPβ and C/EBPα. 3T3-L1 cells were transfected with siRNA against USP10 or treated with spautin-1 for 48 h. **c** His-tag pull-down assays demonstrating a direct interaction between USP10 and C/EBPβ. **d** Spautin-1 dose response (endogenous ubiquitin): increased polyubiquitin-conjugated C/EBPβ with increasing spautin-1 at 24 h vs. vehicle (DMSO). **e** Spautin-1 dose response (HA-ubiquitin overexpression): increased C/EBPβ polyubiquitination under ubiquitin-overexpression conditions. Where indicated, cells were co-transfected with HA-ubiquitin; C/EBPβ ubiquitination was assessed by denaturing C/EBPβ immunoprecipitates followed by anti-HA/anti-ubiquitin immunoblotting. MG132 (10 µM) was used for 8 h, where specified, to accumulate the ubiquitinated species. **f** USP10 knockdown: siUSP10 (48 h) enhances C/EBPβ polyubiquitination and reduces the steady-state C/EBPβ relative to that of the siControl (scRNA). **g** Catalytic dependence (ubiquitination): overexpressed USP10^WT^ decreases C/EBPβ ubiquitination, whereas USP10^C424S^ (catalytically inactive) fails to do so. **h** Catalytic dependence (steady-state protein): USP10^WT^ restores C/EBPβ abundance in a dose-dependent manner, while USP10^C424S^ does not; β-actin served as the loading control. (i) Quantification: densitometric summary of C/EBPβ ubiquitination (panels **a**–**d**) and steady-state C/EBPβ (panel e); data were normalized to the inputs/β-actin and expressed relative to the control (= 1.0). Data are presented as the mean ± SD from *n* = 3 independent experiments. Statistics: one-way ANOVA with Dunnett’s post hoc vs. control or a two-tailed unpaired *t*-test was used for specified pairwise comparisons. Significance: *p < 0.05. USP, ubiquitin-specific peptidase; DMSO, dimethyl sulfoxide; ANOVA, analysis of variance; SD, standard deviation

To identify which factor was directly regulated by USP10, we performed endogenous co-immunoprecipitation (co-IP) for key candidates. C/EBPβ and C/EBPα were both co-immunoprecipitated with USP10 in HEK293FT (Fig. 4a; Fig. S4a) and in 3T3-L1 (Fig. S5b–c) cells. In contrast, Fig. S5a demonstrates that there was no reproducible USP10 interaction with the lipogenic effectors PPARγ, FASN, or FABP4.

We next examined whether USP10 stabilizes C/EBPβ by opposing proteasome-mediated turnover. When the cells were treated with spautin-1 or transfected with siUSP10, the addition of the proteasome inhibitor MG132 restored C/EBPβ abundance relative to that of the untreated conditions, whereas C/EBPα was not notably rescued (Fig. 4b; Fig. S4b and S5d). Moreover, His-tag pull-down assays confirmed that USP10 bound directly to C/EBPβ (Fig. 4c), but not to C/EBPα (Fig. S4c). Cycloheximide-chase experiments (3–12 h) demonstrated that either spautin-1 treatment or USP10 knockdown accelerated the degradation of C/EBPβ relative to that of the controls, which is consistent with a shortened half-life (Fig. S4d–e; Fig. S5e–f).

The ubiquitination assays provided direct mechanistic support, where the pharmacological inhibition of USP10 with spautin-1 treatment increased the endogenous expression levels of polyubiquitin-conjugated C/EBPβ in a dose-dependent manner (Fig. 4d; Fig. S6a) as well as under ubiquitin-overexpression conditions (Fig. 4e; Fig. S6b). Similarly, siRNA-mediated USP10 knockdown enhanced C/EBPβ polyubiquitination and reduced the steady-state protein levels (Fig. 4f; Fig. S6c). Notably, the catalytically inactive mutant USP10^C424S^ failed to reduce C/EBPβ ubiquitination or rescue its protein levels, whereas the wild-type USP10^WT^ decreased C/EBPβ ubiquitination (Fig. 4g; Fig. S6d) and restored its abundance in a dose-dependent manner (Fig. 4h–i; Fig. S6e–f). Together, these results demonstrate that USP10 binds directly to C/EBPβ and maintains its stability through deubiquitination, thereby establishing a DUB-activity-dependent mechanism of C/EBPβ regulation.

### Spautin-1 improves systemic metabolic fitness in HFD-induced obese mice

**Fig. 5:**
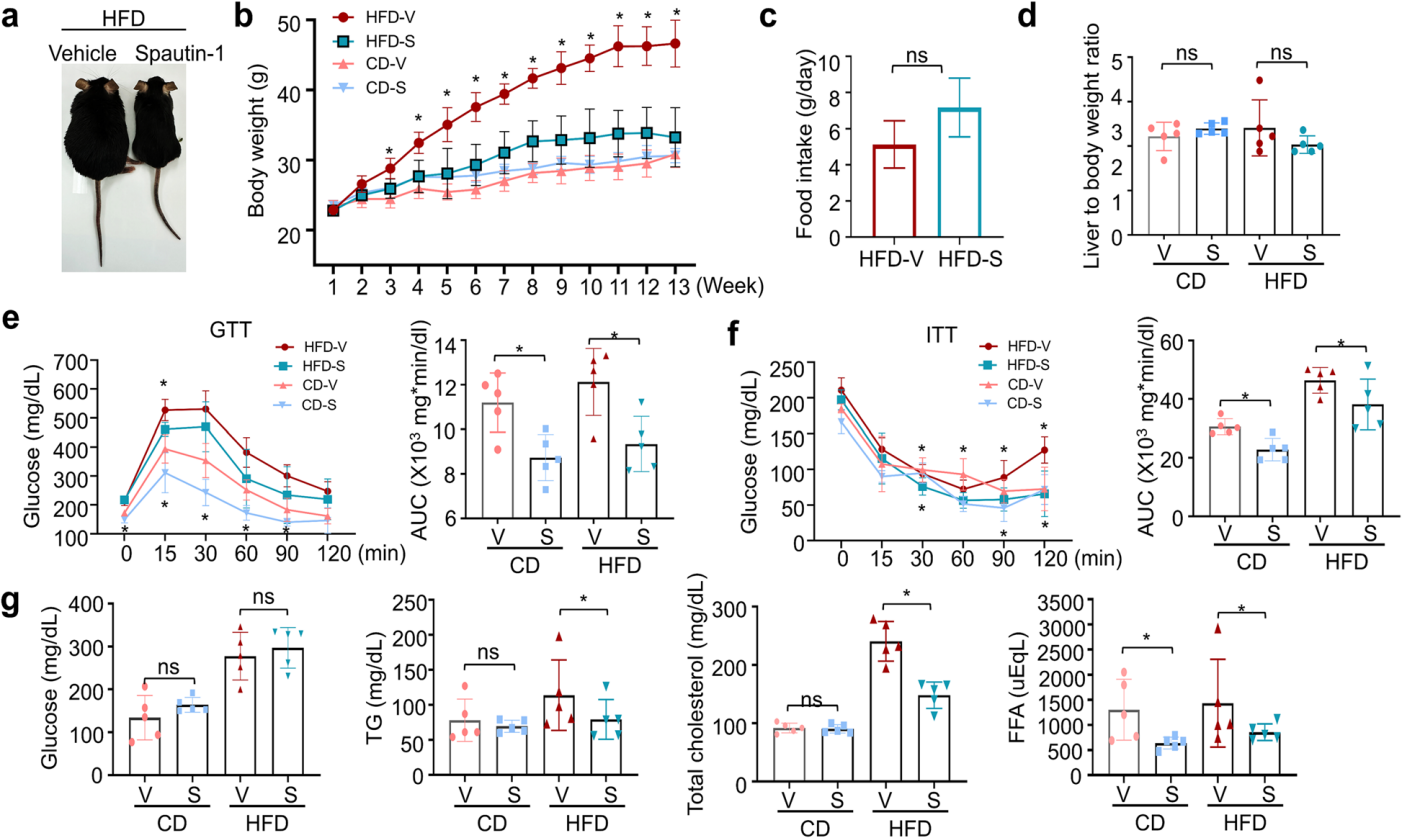
Spautin-1 improves metabolic fitness in diet-induced obesity. Mice were maintained on CD or HFD for 14 weeks, and phenotypic endpoints were assessed after week 14. Spautin-1 (50 mg/kg) or vehicle (DMSO) was administered intraperitoneally three times per week beginning in week 3 (after 2 weeks on HFD); CD controls received the vehicle. **a** Representative images of mice from each group at the endpoint. **b** Body-weight trajectories (weekly) for the CD + vehicle, CD + spautin-1, HFD + vehicle, and HFD + spautin-1 groups. **c** Average daily food intake in the HFD cohorts (weeks 3–14). **d** Liver-to-body weight ratio at study endpoint. **e** Glucose tolerance test: fasting for 15 h, followed by 1 g/kg glucose i.p.; blood glucose levels were measured at 0, 15, 30, 60, 90, and 120 min; curves and AUC shown. **f** Insulin tolerance test: fasting for 6 h, followed by 1 IU/kg insulin i.p.; glucose levels were measured at the indicated times; curves and AUC are shown. **g** Serum biochemistry at study endpoint: fasting glucose, triglycerides, total cholesterol, and FFAs. Data are presented as the mean ± SD from five mice/group. Statistics: longitudinal curves were analyzed by two-way repeated-measures ANOVA with Sidak’s post hoc; end-point comparisons within diet groups were analyzed by a two-tailed unpaired *t-*test (or one-way ANOVA with Dunnett’s, where appropriate). Significance: *p < 0.05 vs. vehicle within the same diet. CD, chow diet; HFD, high-fat diet; DMSO, dimethyl sulfoxide; AUC, area under the curve; FFA, free fatty acid; SD, standard deviation; ANOVA, analysis of variance

To assess the anti-obesity efficacy of spautin-1 in vivo, 7-week-old C57/B6 mice were placed on an HFD for 14 weeks; beginning in week 3, spautin-1 was administered intraperitoneally (i.p.) three times per week, with vehicle-treated HFD and normal chow diet (CD) cohorts as the controls. Spautin-1 (S) significantly diminished body weight gain in the HFD-fed mice relative to the vehicle (V), whereas the body weight in the CD-fed mice was unaffected (Fig. 5a–b). These effects occurred without changes in food intake and with no difference in the liver-to-body-weight ratio (Fig. 5c–d), thus indicating that weight attenuation was not attributable to hypophagia or hepatomegaly.

Spautin-1 treatment improved the metabolic parameters, including glucose tolerance and insulin sensitivity (Fig. 5e–f), and reduced the levels of circulating triglycerides, total cholesterol, and free fatty acids (FFAs) (Fig. 5g). Taken together, these results reveal that spautin-1 ameliorates obesity-induced metabolic dysfunction and improves systemic metabolic fitness in vivo.

### Spautin-1 limits adipose tissue expansion and suppresses adipogenic, lipogenic, and inflammatory processes in vivo

**Fig. 6:**
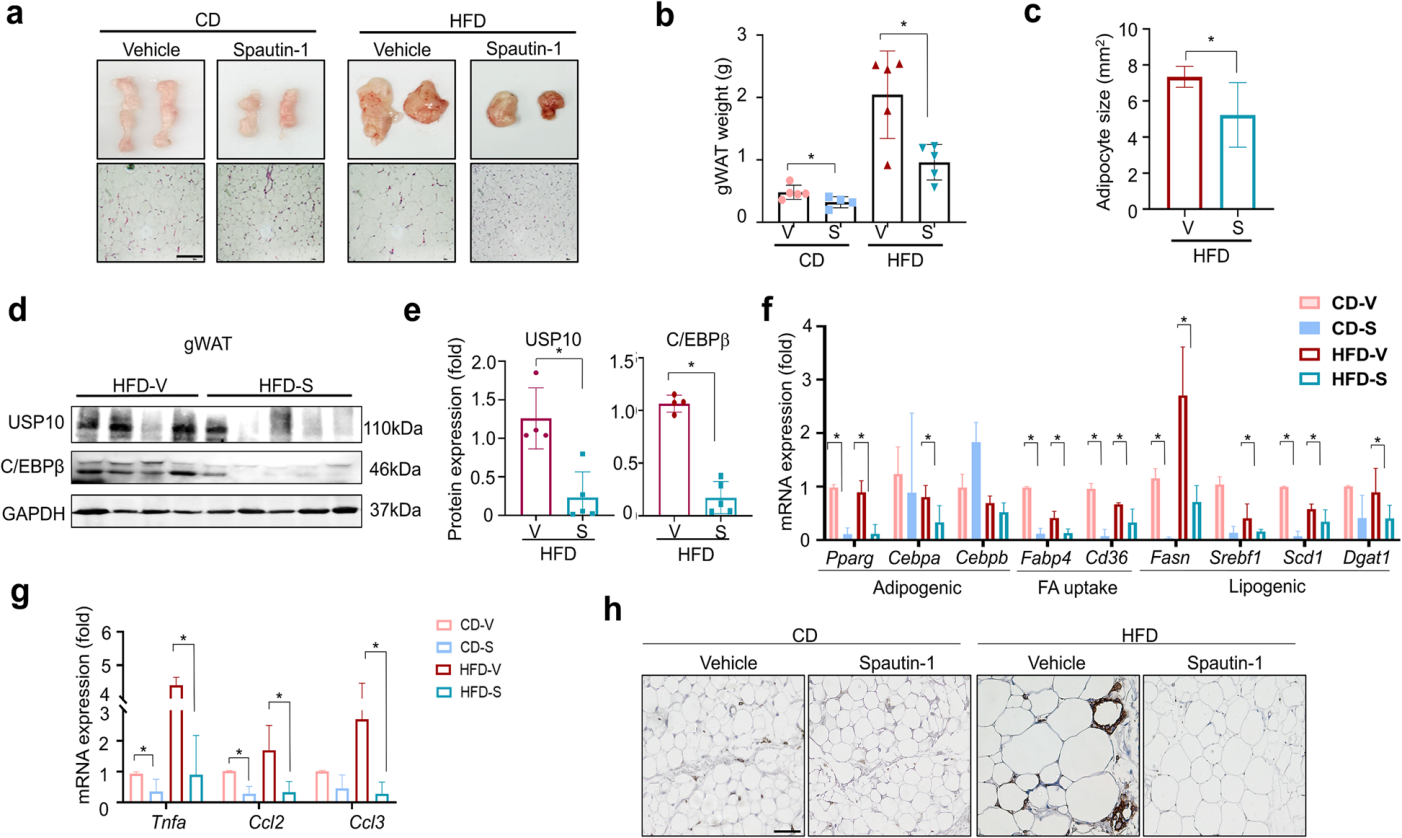
Spautin-1 reduces adiposity, adipocyte size, and pro-adipogenic/pro-inflammatory programs in gWAT. Mice were maintained on CD or HFD for 14 weeks, and the phenotypic endpoints were assessed after week 12. Dosing as in Fig. 5 (Spautin-1, 50 mg/kg, i.p., three times per week from week 3). **a**–**b** gWAT pad weight at study endpoint for the CD and HFD cohorts (iWAT pad weight in Fig. S7a–b). Representative H&E-stained sections of gWAT at study endpoint (scale bar = 100 µm). **c** Adipocyte area was quantified from the H&E-stained sections using ImageJ/Fiji (Adiposoft/fixed-threshold workflow); ≥ 300 adipocytes were assessed per mouse; blinded analysis; per-mouse means are plotted. **d**–**e** Immunoblot analysis of USP10 and C/EBPβ in gWAT with densitometric quantification; GAPDH served as the loading control. Signals were normalized to that of GAPDH and expressed relative to the vehicle (= 1.0) within each diet. **f** RT-qPCR of adipogenic (*Pparg*, *Cebpa*, and *Cebpb*), fatty acid uptake (*Fabp4* and *Cd36*), and lipogenic transcripts in gWAT (*Fasn*, *Srebf1*, *Scd1*, and *Dgat1*); β-actin served as the reference; data were expressed relative to the vehicle within diet. **g** RT-qPCR of inflammatory genes (*Tnfa*, *Ccl2*, and *Ccl3*) in gWAT; β-actin served as the reference. (See Fig. S7 for concordant data in iWAT.) **h** F4/80 immunostaining of gWAT showing crown-like structures (CLS; scale bar = 100 µm). Data are mean ± SD from five mice/group. Statistics: within-diet comparisons were performed using a two-tailed unpaired *t-*test (or a one-way ANOVA with Dunnett’s post hoc test, where applicable). Significance: *p < 0.05 vs. vehicle within the same diet. gWAT, gonadal white adipose tissue; CD, chow diet; HFD, high-fat diet; iWAT, inguinal white adipose tissue; H&E, hematoxylin and eosin; USP, ubiquitin-specific peptidase; RT-qPCR, real time-quantitative polymerase chain reaction; SD, standard deviation; ANOVA, analysis of variance

Under the same in vivo treatment regimen, spautin-1 reduced the overall adiposity and constrained depot expansion. The gWAT and iWAT weights were lower in the spautin-1–treated mice compared with those of vehicle controls in the HFD- and CD-fed cohorts (Fig. 6a; Fig. S7a), and in the HFD-fed mice, histology revealed smaller adipocytes and reduced lipid droplet area (Fig. 6b–c; Fig. S7b–c). In the CD-fed young adult mice, de novo adipocyte progenitor/precursor cell (APC)-derived adipogenesis was minimal; instead, spautin-1 primarily reduced adipocyte hypertrophy, which occurred concomitantly with the USP10–C/EBPβ–dependent suppression of lipogenic programs (e.g., *Pparg*, *Fasn*, and *Fabp4*), resulting in decreased adipose mass (Fig. 6b–c; Fig. S7b–c). Target engagement was confirmed in the gWAT, where the protein levels of USP10 and its downstream effector C/EBPβ were significantly decreased in the HFD-fed mice (Fig. 6d–e). At the molecular level, spautin-1 suppressed adipogenic/lipogenic gene expression, including *Pparg*, *C/ebpa*, *C/ebpb*, *Fabp4*, *Cd36*, *Fasn*, *Srebf1*, *Scd1*, and *Dgat1* in the gWAT (Fig. 6f), with concordant decreases in *Pparg*, *Fasn*, *Fabp4*, *Srebf1*, and *Cd36* in the iWAT (Fig. S7d).

Inflammation was markedly diminished in the spautin-1–treated adipose tissue. Spautin-1 lowered the HFD-elevated inflammatory transcripts (*Tnfa, Ccl2*, and *Ccl*3) in both depots (Fig. 6g; Fig. S7e). Consistently, F4/80 immunohistochemistry and crown-like structure (CLS) quantification in the gWAT revealed abundant CLSs in the vehicle controls but no detectable CLSs in the spautin-1–treated mice (Fig. 6h). Collectively, spautin-1 reduces fat-pad mass and adipocyte size while downregulating the adipogenic/lipogenic and inflammatory pathways, accompanied by target engagement in adipose tissue.

### Spautin-1 reduces hepatic lipid accumulation and inflammation through adipose–liver crosstalk

**Fig. 7:**
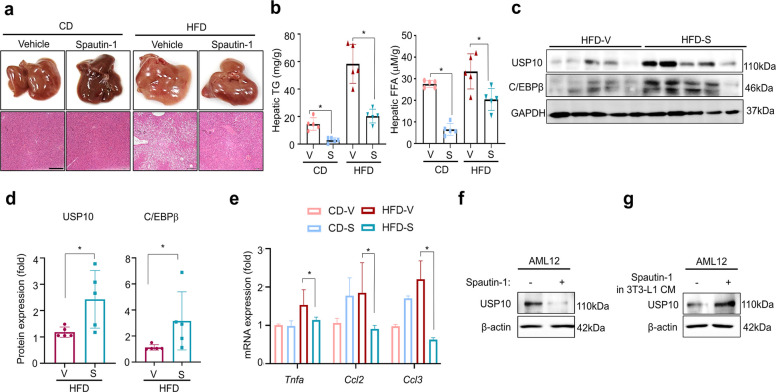
Spautin-1 reduces the hepatic lipid burden and inflammatory condition in diet-induced obesity. Mice were maintained for 14 weeks on CD or HFD and dosed as in Fig. 5 (Spautin-1, 50 mg/kg, i.p, three times per week from week 3). **a** Representative gross liver images and H&E-stained sections at study endpoint (scale bar = 100 µm). **b** Hepatic triglycerides and FFAs were biochemically quantified in the liver homogenates; values were normalized to tissue weight and expressed relative to the vehicle within the diet. **c**-**d** Immunoblot analysis of USP10 and C/EBPβ in gWAT with densitometric quantification; GAPDH served as the loading control. Signals were normalized to that of GAPDH and expressed relative to the vehicle (= 1.0) within each diet. **e** RT-qPCR of hepatic inflammatory transcripts (*Tnfa*, *Ccl2*, and *Ccl3*); β-actin served as the reference gene; data are expressed relative to the vehicle within the diet. **f** AML-12 cells were incubated with spautin-1 or (**g**) treated with CM of 3T3-L1 cells for 24 h. β-actin served as the loading control. Data are presented as the mean ± SD from five mice/group. Statistics: within-diet comparisons were performed using a two-tailed unpaired *t-*test (or a one-way ANOVA with Dunnett’s post hoc test for multi-group comparisons, where applicable). Significance: *p < 0.05 vs. vehicle within the same diet. CD, chow diet; HFD, high-fat diet; H&E, hematoxylin and eosin; FFA, free fatty acid; gWAT, gonadal white adipose tissue; USP, ubiquitin-specific peptidase; RT-qPCR, real-time quantitative polymerase chain reaction; CM, conditioned media; SD, standard deviation; ANOVA, analysis of variance

Given the liver’s central role in systemic lipid metabolism and metabolic-associated steatotic liver disease (MASLD), we evaluated hepatic responses to spautin-1 in vivo. Histological analysis revealed fewer hepatic lipid droplets in the spautin-1–treated HFD mice compared with those of the vehicle-treated mice (Fig. 7a), with corresponding reductions in the hepatic triglyceride and FFA content in the CD- and HFD-fed cohorts (Fig. 7b). Under CD conditions, hepatic lipid content was reduced along with a lower adipose mass (Fig. 7a–b).

This phenotype likely reflects reduced adipocyte hypertrophy and diminished lipogenic signaling via the USP10–C/EBPβ axis in adipose tissue, thereby lowering the fatty acid flux and inflammatory signals to the liver. Interestingly, the USP10 and C/EBPβ protein levels were increased in the livers of spautin-1–treated HFD mice (Fig. 7c–d), which is opposite to the pattern observed in the adipose tissue but consistent with reports linking hepatic USP10 upregulation with steatosis improvement [22]. Nevertheless, spautin-1 reduced hepatic inflammation, significantly lowering the tumor necrosis factor alpha (*Tnfa*) and C–C motif chemokine 2 and 3 (*Ccl2* and *Ccl3*) transcripts (Fig. 7e).

To assess the inter-tissue communication, we exposed primary hepatocytes to conditioned media (CM) derived from 3T3-L1 adipocytes differentiated with or without spautin-1 treatment. The direct spautin-1 treatment of hepatocytes reduced the USP10 protein levels (Fig. 7f), whereas hepatocytes treated with CM from spautin-1–treated adipocytes exhibited increased USP10 protein expression (Fig. 7g). Together, these findings indicate that spautin-1 lowers the hepatic lipid burden and inflammation via adipose–liver crosstalk and the tissue-specific regulation of USP10.

## Discussion

This study provides compelling evidence that spautin-1 exerts anti-obesity effects in part by inhibiting USP10 and destabilizing C/EBPβ in adipose tissue. In so doing, it reframes spautin-1 from an autophagy-linked compound into a DUB-directed modulator of adipocyte biology and highlights USP10 as a promising target for metabolic intervention. Our results establish that USP10 plays a pivotal role in adipogenesis and lipid accumulation, with its pharmacological inhibition attenuating these processes through C/EBPβ destabilization.

As spautin-1 targets USP10 and USP13 [12], we first assessed which enzyme is most relevant to adipogenesis. Analysis of publicly available RNA-sequencing datasets (GSE213058 and GSE235696) revealed that USP10, but not USP13, was elevated in adipose tissue from adults with obesity compared with that of lean controls, with a bias toward VAT over SAT. Consistently, in mouse adipose tissue, the mRNA and protein levels of USP10 were increased in obesity, whereas USP13 revealed a high Ct by RT-qPCR and was undetectable by immunoblot, which aligns with FANTOM5 (FF:10010-101C1). In 3T3-L1 differentiation, USP10 progressively increased, whereas USP13 only appeared near terminal differentiation. This temporal stratification places early C/EBPβ–FASN lipogenic modules upstream of late cytoskeletal/extracellular matrix (ECM) programs [23]. Functionally, siUSP10 markedly impaired differentiation and reduced the levels of PPARγ, C/EBPα, C/EBPβ, FASN, and FABP4, whereas siUSP13 produced only mild effects. Collectively, USP10, rather than USP13, sustains adipogenesis and lipid accumulation as a critical DUB.

Mechanistically, USP10 engages with C/EBPβ and preserves its stability through deubiquitination. Co-IP and His-tag pull-down showed selective binding to C/EBPβ and not C/EBPα. Cycloheximide-chase revealed a shortened C/EBPβ half-life with spautin-1 treatment or USP10 knockdown, and ubiquitination assays demonstrated increased polyubiquitinated C/EBPβ when USP10 was inhibited. Rescue with USP10^WT^, but not USP10^C424S^, confirmed that USP10 was required for DUB activity. Together, these data define a USP10 → C/EBPβ axis that is necessary for efficient adipogenic commitment. Moreover, spautin-1 is known as an autophagy inhibitor that acts on the Vps34/Beclin-1 complex via USP10/USP13, thereby reducing Beclin-1/LC3B-II and flux [12]. In preadipocytes, autophagy is transiently required early on, and its attenuation during induction can suppress adipogenesis. However, in mature adipocytes, autophagy supports quality control, and chronic blockades can induce pro-inflammatory conditions [24]. In mice fed an HFD, spautin-1 treatment lowered the levels of inflammatory cytokines and improved insulin sensitivity, thus arguing against a net “autophagy-loss” phenotype and supporting autophagy-independent contributions, primarily via USP10-dependent C/EBPβ destabilization. From a pathophysiological standpoint, adult obesity reflects hypertrophic lipid loading and depot-biased inflammation [24]. Beyond early commitment, C/EBPβ remains active in mature adipose tissue, which supports the lipogenic (FASN, SREBF1, and CD36) and pro-inflammatory (TNFA, CCL2, and CCL3) programs [25, 26]. Thus, reducing but not ablating C/EBPβ is predicted to reduce triglyceride synthesis and chemokine/cytokine output, limit macrophage recruitment, improve adipose insulin signaling, and reduce lipotoxic FFA spillover [26, 27]. In HFD-fed mice, spautin-1 attenuated weight gain without affecting food intake, improved glucose tolerance, lowered circulating triglycerides, cholesterol, and FFAs, and reduced the fat-pad mass and adipocyte size, with the concordant downregulation of lipogenic/inflammatory transcripts and target engagement in gWAT (decreased USP10 and C/EBPβ). The body weight in CD-fed mice was unchanged, thereby indicating diet-context–dependent efficacy. There are alternative mechanisms that may potentially contribute to the observed improvement in systemic metabolic fitness with spautin-1 treatment. Specifically, spautin-1 enhances energy expenditure or reduces intestinal lipid absorption; therefore, further investigation into these pathways is warranted.

Therapeutically, depot context matters, as USP10 was elevated in human VAT, and the pharmacodynamic effects were preferential in murine gWAT. As VAT confers disproportionate risk, selectively reducing lipid accrual and inflammatory conditions in visceral depots may yield substantial benefit while preserving SAT’s buffering capacity [28]. Accordingly, USP10 is VAT-enriched and a drug-targetable molecule; attenuating the USP10–C/EBPβ axis in visceral depots limits hypertrophy and inflammatory output, reduces lipotoxic liver flux, and improves the systemic metabolic indices without altering intake, which suggests patient enrichment (high VAT burden and elevated adipose USP10) and visceral-selective delivery.

A tissue-specific divergence emerged in the liver, where spautin-1 increased hepatic levels of USP10 (and C/EBPβ) but reduced hepatic levels of triglycerides and FFA, as well as the inflammatory transcripts. Similar genetic–pharmacology divergences have been reported (e.g., USP30) [29] in studies linking liver USP10 with liver kinase B1 (LKB1) and sirtuin 6 (SIRT6) [30]. Context dependence is well documented for other molecules as well (e.g., sirtuin (SIRT1), mammalian target of rapamycin complex 1(mTORC1), etc.) [31–34]. Our hepatocyte experiments provide mechanistic insights consistent with inter-organ communication. Adipose-derived CM upregulated hepatic USP10, whereas direct spautin-1 exposure downregulated it, thus implicating a balance between cell-autonomous drug action and non-cell-autonomous adipose–liver signals (e.g., adipokines and FFA flux) [35–38]. Taken together, these considerations emphasize the need to parse tissue-selective pharmacodynamics and adipose–liver crosstalk when targeting DUBs.

Importantly, the pharmacology of spautin-1 extends beyond USP10 inhibition, which might contribute to its anti-obesity efficacy and yield outcomes that do not fully mirror the tissue-selective USP10 suppression. Spautin-1 was originally identified as an autophagy inhibitor that promotes Vps34/Beclin-1 complex degradation via USP10/USP13, with secondary effects on the homeostasis of p53 [12]. It has also been reported to inhibit mitochondrial complex I and suppress the unfolded-protein response under metabolic stress [5]. These stress–response pathways shape cellular energetics and inflammation across tissues and likely function alongside the USP10 → C/EBPβ adipocyte program to produce this phenotype in vivo. Therapeutically, depot context remains crucial, as visceral adiposity confers a disproportionate risk, and the VAT/SAT distribution is strongly associated with the outcomes [8–10]. In this setting, USP10 is a VAT-relevant, drug-targetable molecule. The pleiotropy of spautin-1 argues for the development of next-generation, depot-aware analogs or rational combinations to capture desired adipose effects while minimizing extra-adipose adverse effects.

This study had some limitations. Specifically, mechanistic assays relied on 3T3-L1 and HEK293FT cells; therefore, adipocyte- and hepatocyte-specific genetic models (loss and rescue) would clarify tissue causality in vivo. Because spautin-1 targets USP10 and USP13 and is linked to autophagy, broader selectivity and depot-resolved pharmacokinetics/pharmacodynamics are warranted. Although our human analyses employed public transcriptomic datasets and not direct patient samples, protein-level validation in paired VAT/SAT could strengthen the translation of the results. Finally, we did not distinguish between the C/EBPβ isoform-specific effects (LAP vs. LIP). Addressing these limitations could help elucidate how the USP10-directed regulation of C/EBPβ integrates with autophagy and stress–response networks to improve metabolism.

In conclusion, spautin-1 suppresses adipogenesis and lipid accumulation by inhibiting USP10 and destabilizing C/EBPβ, while improving the systemic metabolic indices in HFD mice. These findings position spautin-1 as a pharmacological lead and motivate the optimization of spautin-1–based analogs and mechanism-guided combinations for selective adipose remodeling with systemic benefits.

## Materials and methods

### Reagents and antibodies

Spautin-1 (TargetMol, T1937) was dissolved in DMSO at 100 mM (stock) and stored at − 20 °C, and working dilutions were prepared fresh in culture media with a final DMSO concentration of ≤ 0.1% (vehicle control). MG132 (proteasome inhibitor; TargetMol, T2154, 10–15 μM) and cycloheximide (Selleckchem, S7418, 100 μM) were used. The antibodies used in this study are listed in Supplementary Table S2. Bradford reagents, enhanced chemiluminescence (ECL) kits, and polyvinylidene fluoride (PVDF) membranes were obtained from Sigma Aldrich (B6916), Thermo Scientific (SL368236), and Millipore (IPVH00010), respectively.

### Cell lines and authentication

3T3-L1 preadipocytes (ATCC, CL-173) and HEK293 cells (ATCC, CRL-1573) were obtained from ATCC and used between passages 4 and 19. The cells were tested for mycoplasma contamination using the TaKaRa PCR Mycoplasma Detection Set (TaKaRa, 6601).

### Cell culture

3T3-L1 preadipocytes were cultured in high-glucose Dulbecco’s Modified Eagle’s Medium (DMEM, Welgene, LM001-014.5 g/L) with 10% calf serum (Gibco, 16170078) and 1% penicillin–streptomycin (P/S, Gibco, 15240062) at 37 °C and under 5% CO_2_ in a humidified incubator. The HEK293FT cells were maintained in DMEM with 10% fetal bovine serum (FBS, Gibco, 11570506) and 1% P/S under the same conditions. AML-12 mouse hepatocytes were cultured in DMEM/F-12 (Gibco, 11330032) with 10% FBS and 1% P/S, as previously described [39]. All cell lines were routinely verified as mycoplasma-negative and used within the recommended passage ranges.

### Adipocyte differentiation

3T3-L1 preadipocytes were seeded at a cell density of 2.0 × 10^5^ cells/well in 6-well plates and cultured at 37 °C under 5% CO_2_ conditions until reaching confluence. Day 0 was defined as two days post-confluence. Differentiation was initiated by replacing the medium with DMEM, 10% FBS, and 1% penicillin/streptomycin containing an MDI cocktail (3-Isobutyl-1-methylxanthine (IBMX), Sigma Aldrich, 0.5 mM I7018-100MG, 1 µM dexamethasone, and 10 µg/mL of insulin) for 48 h. On Day 2, the cells were switched to DMEM, containing 10% FBS and 10 µg/mL of insulin only. The medium was changed every 48 h until Day 6, as described previously [40]. Successful induction was verified, and the experimental timeline is presented in Fig. S1a. Lipid accumulation became microscopically detectable by Day 4 and increased by Day 6 (Fig. S1b). For temporal molecular profiling, cells were collected at Days 0, 2, 4, and 6 for RNA and protein analyses; see the qPCR and immunoblot sections. ORO staining and quantification were performed as detailed in the “Oil Red O staining” section. Unless otherwise stated, experiments were performed with at least three biological replicates.

### Oil Red O (ORO) staining

On Day 6 of differentiation, Dulbecco’s phosphate-buffered saline (DPBS, Welgene, LB001-02) was used to wash the differentiated 3T3-L1 cells, and the cells were then incubated in 10% neutral-buffered formalin (Georgiachem, F291583O) for 30 min at room temperature. These cells were rinsed with distilled water, followed by 60% isopropanol (Supelco, I1322734 404), and then air-dried. An ORO working solution was prepared by diluting ORO stock (Sigma Aldrich, O0625, 0.35 g/100 mL in isopropanol) to 60% with distilled water and filtering through a 0.22-µm membrane. The dried cells were stained with the prepared ORO working solution for 60 min, washed three times with distilled water, and photographed under identical settings. Bound dye was eluted in 100% isopropanol for 10 min, and the absorbance was measured at 500 nm using a plate reader. Isopropanol blank readings were subtracted from the eluted dye readings, and values were normalized to that of the vehicle control. Representative images and corresponding quantification graphs were prepared, with the data derived from at least two independent experiments.

### Cell viability

3T3-L1 cells were seeded in 12-well plates and treated with spautin-1 or vehicle (0.1% DMSO) for 72 h. The viability was measured using EZ-Cytox (Daeil Lab Service, EZ-1000) as per the manufacturer’s protocol, and absorbance was measured at 450 nm (minus the background). Values were normalized to that of the vehicle (= 100%), and data are presented from ≥ 2 independent experiments with replicate wells.

### RNA isolation and quantitative PCR

Total RNA was extracted using an RNA-lysis reagent (Intron, 17061), treated with DNase I, and 1 µg was reverse-transcribed with ReverTra Ace qPCR RT Master Mix (TOYOBO, FSQ-201). RT-qPCR was performed with TB Green Premix Ex Taq (Takara, AO9R007) on an ABI real-time system (Applied Biosystems) using primers that are presented in Table S1. β-Actin served as the reference gene, and the relative expression was calculated from technical triplicates and ≥ 3 biological replicates.

### Transfection of siRNAs and plasmid DNA

For target knockdown, 3T3-L1 cells were transfected with 20 nM of siUSP10 or siUSP13 or a non-targeting siControl (scRNA) using Lipofectamine RNAiMAX (Invitrogen, 13778075) in an antibiotic-free medium. The siRNA sequences are listed in Table S2. After 24 h, the cells were switched to maintenance medium (DMEM + 10% calf serum) and either harvested 48–72 h later for knockdown verification (qPCR and immunoblot) or induced to differentiate as per the adipogenesis protocol. For the overexpression and ubiquitination assays, the cells were transfected with pcDNA-C/EBPβ, pCMV-FLAG-USP10, and/or pSG5-HA–Ubiquitin using Lipofectamine 2000 (Invitrogen, 11668500). An empty vector served as a control. Cells were collected 24–48 h post-transfection for downstream analyses. All transfections were performed in at least three independent experiments.

### Measurement of the cell number during MCE

3T3-L1 preadipocytes were seeded in 6-well plates and transfected with siUSP10, siUSP13, or the non-targeting siControl (scRNA) using Lipofectamine RNAiMAX, as described previously [41]. The siRNA sequences are listed in Table S2. At 24 h and 48 h post-transfection, the cells were detached with 0.05% trypsin–EDTA (Gibco, 2085271), resuspended in PBS + 0.5% bovine serum albumin (BSA; Bovogen, BSAS 0.1), and counted on a NanoEnTek automated counter with trypan blue exclusion as per the manufacturer’s instructions. Cell counts from triplicate wells were averaged and normalized to those of the siControl (= 100%). Data are presented from at least two independent experiments.

### Western blot analysis

Cells and tissues were lysed on ice in RIPA buffer (BioSesang, RC2002-050–00) supplemented with protease/phosphatase inhibitors (1 mM Na₃VO₄, 1 mM NaF, and a protease inhibitor cocktail; Gene de-pot, 08031622), as described previously [42]. The lysates were incubated on ice for 20 min and centrifuged at ~ 16,000 × g for 20 min at 4 °C. Protein concentration was determined using the Bradford assay. Samples were mixed with Laemmli SDS sample buffer, including β-mercaptoethanol (Sigma, M6250), and boiled for 5 min at 95 °C. Equal amounts of protein (20–40 μg) were separated by sodium dodecyl sulfate–polyacrylamide gel electrophoresis (SDS-PAGE) and transferred to polyvinylidene fluoride (PVDF) membranes. The membranes were blocked with 5% BSA in TBST for phospho-targets or 5% nonfat milk in TBST for total proteins for 1 h at room temperature and incubated with the primary antibodies overnight at 4 °C. These were then washed three times for 10 min each in TBST, incubated with horseradish peroxidase (HRP)-conjugated secondary antibodies for 1 h at room temperature, and developed by electrochemiluminescence (ECL) on a FUSION SOLO imager (Vilber). The band intensities were quantified in ImageJ and normalized to those of β-actin or GAPDH as loading controls. Antibodies and their manufacturers are listed in Table S3.

### Co-IP

Cells were lysed in IP buffer (50 mM Tris–HCl at pH 7.5, 150 mM NaCl, and 1% NP-40 or Triton X-100, 0.5% sodium deoxycholate, 0.1% SDS, and 2 mM EDTA) supplemented with protease/phosphatase inhibitors. Lysates (1–2 mg total protein) were centrifuged at ~ 16,000 × g for 20 min at 4 °C, quantified, equalized, and precleared with Protein A/G agarose (Santa Cruz, sc-2003) for 30 min at 4 °C. The supernatants were incubated overnight at 4 °C with anti-USP10 or anti-C/EBPβ (2–4 µg) or species-matched IgG (negative control) under constant agitation, followed by capture with Protein A/G beads for 1–2 h at 4 °C. These beads were washed 3–5 times with IP buffer, and the bound proteins were eluted in 2X Laemmli/SDS sample buffer and boiled for 5–10 min at 95 °C. Inputs (5–10%) and immunoprecipitates were analyzed by immunoblotting, as described previously [42].

### His-tag pull-down

Purified His–USP10 (Creative BioMart, NY, USA; cat. USP10-12H, 2 μg) was incubated with GST–C/EBPb (Abnova, H00001051-P01) or GST–C/EBPa (Abnova, H00001051-P01) in a binding buffer (20 mM HEPES–KOH at pH 7.5, 300 mM NaCl, 1 mM EDTA, 1% Triton X-100, 0.1% sodium deoxycholate, and 0.02% SDS) at 4 °C for 2 h with constant agitation. Complexes were captured with Ni–NTA agarose (QIAGEN, 124114376) as per the manufacturer’s instructions and then washed three times with binding buffer and eluted in 2 × Laemmli sample buffer at 95 °C for 5 min. Inputs (10%) and pull-down fractions were analyzed by immunoblotting using anti-USP10 and anti-GST antibodies to assess direct binding. Negative controls (His–USP10 only; GST–C/EBPβ only) were processed in parallel.

### Ubiquitination assay

Cells were transfected with HA-ubiquitin and the indicated constructs. To preserve the ubiquitin conjugates, all buffers contained 5 mM N-ethylmaleimide (NEM, Pierce, 23030) with protease/phosphatase inhibitors. To denature the immunoprecipitates, the cells were lysed in PBS with 1% SDS, boiled for 5 min, and diluted 10 × with non-denaturing lysis buffer (20 mM Tris–HCl, pH 7.5, 150 mM NaCl, 1% Triton X-100, and 1 mM EDTA). The viscosity was reduced by passing the lysates 3–5 times through a 1-mL syringe needle, incubating for 5 min on ice, and clearing by centrifugation at ~ 16,000 × g for 10 min at 4 °C. Equal amounts of protein were immunoprecipitated with anti-C/EBPβ (2–4 µg) or species-matched IgG (negative control) overnight at 4 °C, captured with Protein A/G agarose for 1–2 h at 4 °C, washed 3–5 times, and eluted in 2 × Laemmli buffer at 95 °C for 5–10 min. Polyubiquitinated C/EBPβ was detected by anti-HA/anti-ubiquitin immunoblotting, and inputs (5–10%) were loaded. For pharmacologic perturbations, spautin-1 (or vehicle) was added 6–12 h before harvest; MG132 (Enzo, BML-PI102, 10 µM) was used where indicated to accumulate the ubiquitinated species. Data are presented from ≥ 3 independent experiments.

Protein stability was assessed by treating the cells with cycloheximide (TargetMol, T1225, 200 µM) under the indicated conditions with or without spautin-1 or siRNA. The cells were collected at 0, 3, 6, 9, and 12 h, lysed for immunoblotting, and the C/EBPβ band intensities were normalized to time 0, and β-actin served as the loading control. Half-lives were estimated by single-exponential decay fitting; results are presented from ≥ 3 independent experiments.

### Animal studies

Six-week-old male C57BL/6 mice (Orientbio, Seongnam, Korea) were acclimated for 1 week (12-h light/dark cycle, 22 ± 2 °C, and 50%–60% humidity) with ad libitum access to food and water, then assigned to a CD or HFD (60% kcal fat, D12492; Research Diets) group for 13 weeks (obese phenotype at 12 weeks as previously reported [37]). After 2 weeks on HFD (start of week 3), the HFD mice were randomized to vehicle (DMSO) or spautin-1 (50 mg/kg) group three times per week until the end of the study. CD controls received the vehicle. Body weight and food intake were recorded weekly. Randomization was computer-generated, and the investigators were blinded to the treatment during data collection and analysis. The exclusion criteria were pre-specified (e.g., > 15% weight loss or injection-site complications), and unless otherwise stated, no animals were excluded. All procedures were reviewed and approved by the Yonsei University Health System IACUC (2022–0339) and strictly complied with institutional and national guidelines.

### Tissue collection, histology, and morphometry

At the endpoint, gWAT, iWAT, and liver were excised, weighed, and either snap-frozen or fixed in 10% neutral-buffered formalin for 24 h before paraffin embedding. Sections (5 µm) were stained with hematoxylin and eosin (H&E) and photographed via bright-field microscopy on a Zeiss LSM 700 system (Oberkochen, Germany). Adipocyte area and lipid droplet size were quantified using ImageJ from ≥ 5 non-overlapping fields per section and ≥ 3 sections per animal, with ≥ 300 adipocytes per animal analyzed by blinded assessment. Hepatic triglycerides and FFAs were quantified in tissue homogenates using enzymatic kits (Dogen Bio, DG-TGC100 and DG-FFA100) according to the manufacturers’ instructions.

### Metabolic phenotyping

For glucose tolerance tests, mice were fasted for 15 h, then injected intraperitoneally with 1 g/kg of D-glucose (Sigma-Aldrich). For insulin tolerance tests, mice were fasted for 6 h and then injected intraperitoneally with 1 IU/kg of insulin (Sigma-Aldrich). Blood was collected via tail vein sampling after cleaning with 70% ethanol, and blood glucose was measured with a handheld glucometer at 0 (baseline), 15, 30, 60, 90, and 120 min post-injection. Glucose measurements were quantified as area under the curve (AUC, trapezoidal method) and, where indicated, normalized to those of the baseline.

### Public dataset analysis

Human adipose RNA-seq data were obtained from GTEx v8 (Adipose–Visceral [Omentum] and Adipose-Subcutaneous) and obesity case–control cohorts from GEO (GSE235696 and GSE213058). Raw counts were processed in R (v4.2.0) using DESeq2 (v1.36.0) with covariates (age, sex, BMI, and batch as available). The expression values were variance-stabilized, and differential expression was determined using Wald tests with Benjamini–Hochberg false discovery rate (FDR) correction (q < 0.05). Depot comparisons (VAT vs. SAT) used paired models when donor-matched samples were available; otherwise, covariate-adjusted models were applied. Plots were generated using ggplot2 (v3.4.0), and the analyses of human transcriptomic datasets employed publicly available de-identified data (GTEx/GEO) and did not require institutional review board approval.

### Statistical analysis

GraphPad Prism was used to statistically analyze the quantified data. An unpaired two-tailed Student’s *t-*test was used to identify the statistical significance of the bar graphs between two groups. *P*-values < 0.05 indicated statistically significant differences.

### Controls and experimental rigor

Vehicle controls matched the highest DMSO used (≤ 0.1%). For genetics, non-targeting siControl (scRNA) and two independent siRNAs per gene were used. For ubiquitination, MG132 served as the positive control for proteasome inhibition. For stability, cycloheximide- and vehicle-only arms were included. For in vivo analyses, vehicle-treated HFD and CD controls were run in parallel. Randomization and blinding procedures are described above, and sample-size calculations and exclusion criteria were pre-specified.

## Supplementary Information

Supplementary Material 1.

## Data Availability

The bioinformatics data supporting the findings of this study are publicly available from the Gene Expression Omnibus under accession GSE235696 and GSE213058. Additional experimental protocols, reagent information, and additional data are available within the article and/or its supplementary materials.

## References

[1] World Health Organization. World health statistics 2024: monitoring health for the SDGs, sustainable development goals. Geneva: WHO; 2024.

[2] Swinburn BA, Kraak VI, Allender S, Atkins VJ, Baker PI, Bogard JR, et al. The global syndemic of obesity, undernutrition, and climate change: the Lancet commission report. Lancet. 2019;393(10173):791–846. 10.1016/S0140-6736(18)32822-8. 30700377 10.1016/S0140-6736(18)32822-8

[3] Ghaben AL, Scherer PE. Adipogenesis and metabolic health. Nat Rev Mol Cell Biol. 2019;20(4):242–58. 10.1038/s41580-018-0093-z. 30610207 10.1038/s41580-018-0093-z

[4] Kim H-Y, Jang H-J, Muthamil S, Shin UC, Lyu J-H, Kim S-W, et al. Novel insights into regulators and functional modulators of adipogenesis. Biomed Pharmacother. 2024;177:117073. 10.1016/j.biopha.2024.117073. 38981239 10.1016/j.biopha.2024.117073

[5] Dragano NRV, Fernø J, Diéguez C, López M, Milbank E. Reprint of: Recent updates on obesity treatments: available drugs and future directions. Neuroscience. 2020;447:191–215. 10.1016/j.neuroscience.2020.08.009. 33046217 10.1016/j.neuroscience.2020.08.009

[6] Chang SH, Stoll CR, Song J, Varela JE, Eagon CJ, Colditz GA. The effectiveness and risks of bariatric surgery: an updated systematic review and meta-analysis, 2003–2012. JAMA Surg. 2014;149(3):275–87. 10.1001/jamasurg.2013.3654. 24352617 10.1001/jamasurg.2013.3654 PMC3962512

[7] Wang JY, Wang QW, Yang XY, Yang W, Li DR, Jin JY, et al. GLP-1 receptor agonists for the treatment of obesity: role as a promising approach. Front Endocrinol (Lausanne). 2023;14:1085799. 10.3389/fendo.2023.1085799. 36843578 10.3389/fendo.2023.1085799 PMC9945324

[8] Chen S, Liu Y, Zhou H. Advances in the development ubiquitin-specific peptidase (USP) inhibitors. Int J Mol Sci. 2021;22(9):4546. 10.3390/ijms22094546. 33925279 10.3390/ijms22094546 PMC8123678

[9] Ning Z, Guo X, Liu X, Lu C, Wang A, Wang X, et al. USP22 regulates lipidome accumulation by stabilizing PPARγ in hepatocellular carcinoma. Nat Commun. 2022;13(1):2187. 10.1038/s41467-022-29846-9. 35449157 10.1038/s41467-022-29846-9 PMC9023467

[10] Kim MS, Baek J-H, Lee J, Sivaraman A, Lee K, Chun K-H. Deubiquitinase USP1 enhances CCAAT/enhancer-binding protein beta (C/EBPβ) stability and accelerates adipogenesis and lipid accumulation. Cell Death Dis. 2023;14(11):776. 10.1038/s41419-023-06317-7. 38012162 10.1038/s41419-023-06317-7 PMC10681981

[11] Baek JH, Kim MS, Jung HR, Hwang MS, Lee CH, Han DH, et al. Ablation of the deubiquitinase USP15 ameliorates nonalcoholic fatty liver disease and nonalcoholic steatohepatitis. Exp Mol Med. 2023;55(7):1520–30. 10.1038/s12276-023-01036-7. 37394587 10.1038/s12276-023-01036-7 PMC10394025

[12] Liu J, Xia H, Kim M, Xu L, Li Y, Zhang L, et al. Beclin1 controls the levels of p53 by regulating the deubiquitination activity of USP10 and USP13. Cell. 2011;147(1):223–34. 10.1016/j.cell.2011.08.037. 21962518 10.1016/j.cell.2011.08.037 PMC3441147

[13] Tang X, Weng R, Guo G, Wei J, Wu X, Chen B, et al. USP10 regulates macrophage inflammation responses via stabilizing NEMO in LPS-induced sepsis. Inflamm Res. 2023;72(8):1621–32. 10.1007/s00011-023-01768-2. 37436447 10.1007/s00011-023-01768-2

[14] Xu Y-j, Zeng K, Ren Y, Mao C-y, Ye Y-h, Zhu X-t, et al. Inhibition of USP10 induces myeloma cell apoptosis by promoting cyclin D3 degradation. Acta Pharmacol Sin. 2023;44(9):1920–31. 10.1038/s41401-023-01083-w. 37055530 10.1038/s41401-023-01083-w PMC10462714

[15] Kona SV, Kalivendi SV. The USP10/13 inhibitor, spautin-1, attenuates the progression of glioblastoma by independently regulating RAF-ERK mediated glycolysis and SKP2. Biochim Biophys Acta (BBA). 2024;1870(7):167291. 10.1016/j.bbadis.2024.167291. 10.1016/j.bbadis.2024.167291 38857836

[16] Sethi A, Mishra S, Upadhyay V, Dubey P, Siddiqui S, Singh AK, et al. USP10 deubiquitinates and stabilizes CD44 leading to enhanced breast cancer cell proliferation, stemness and metastasis. Biochem J. 2024;481(24):1877–900. 10.1042/BCJ20240611. 39564770 10.1042/BCJ20240611

[17] Feng Z, Ou Y, Deng X, Deng M, Yan X, Chen L, et al. Deubiquitinase USP10 promotes osteosarcoma autophagy and progression through regulating GSK3β-ULK1 axis. Cell Biosci. 2024;14(1):111. 10.1186/s13578-024-01291-9. 39218913 10.1186/s13578-024-01291-9 PMC11367994

[18] Zhang S, Zhang M, Jing Y, Yin X, Ma P, Zhang Z, et al. Deubiquitinase USP13 dictates MCL1 stability and sensitivity to BH3 mimetic inhibitors. Nat Commun. 2018;9(1):215. 10.1038/s41467-017-02693-9. 29335437 10.1038/s41467-017-02693-9 PMC5768685

[19] Wang C, Meng Y, Zhao J, Ma J, Zhao Y, Gao R, et al. Deubiquitinase USP13 regulates glycolytic reprogramming and progression in osteosarcoma by stabilizing METTL3/m(6)A/ATG5 axis. Int J Biol Sci. 2023;19(7):2289–303. 10.7150/ijbs.82081. 37151889 10.7150/ijbs.82081 PMC10158027

[20] Liu X, Balaraman K, Lynch CC, Hebron M, Wolf C, Moussa C. Novel ubiquitin specific protease-13 inhibitors alleviate neurodegenerative pathology. Metabolites. 2021;11(9):622. 10.3390/metabo11090622. 34564439 10.3390/metabo11090622 PMC8467576

[21] Liu H, Zhao Z, Wu T, Zhang Q, Lu F, Gu J, et al. Inhibition of autophagy-dependent pyroptosis attenuates cerebral ischaemia/reperfusion injury. J Cell Mol Med. 2021;25(11):5060–9. 10.1111/jcmm.16483. 33938129 10.1111/jcmm.16483 PMC8178262

[22] Luo P, Qin C, Zhu L, Fang C, Zhang Y, Zhang H, et al. Ubiquitin-specific peptidase 10 (USP10) inhibits hepatic steatosis, insulin resistance, and inflammation through Sirt6. Hepatology. 2018;68(5):1786–803. 10.1002/hep.30062. 29698567 10.1002/hep.30062

[23] Nakamura T, Shiojima S, Hirai Y, Iwama T, Tsuruzoe N, Hirasawa A, et al. Temporal gene expression changes during adipogenesis in human mesenchymal stem cells. Biochem Biophys Res Commun. 2003;303(1):306–12. 10.1016/s0006-291x(03)00325-5. 12646203 10.1016/s0006-291x(03)00325-5

[24] Yoshizaki T, Kusunoki C, Kondo M, Yasuda M, Kume S, Morino K, et al. Autophagy regulates inflammation in adipocytes. Biochem Biophys Res Commun. 2012;417(1):352–7. 10.1016/j.bbrc.2011.11.114. 22155234 10.1016/j.bbrc.2011.11.114

[25] Payne VA, Au WS, Lowe CE, Rahman SM, Friedman JE, O’Rahilly S, et al. C/EBP transcription factors regulate SREBP1c gene expression during adipogenesis. Biochem J. 2009;425(1):215–23. 10.1042/bj20091112. 19811452 10.1042/BJ20091112 PMC2913385

[26] Rahman SM, Janssen RC, Choudhury M, Baquero KC, Aikens RM, de la Houssaye BA, et al. CCAAT/enhancer-binding protein β (C/EBPβ) expression regulates dietary-induced inflammation in macrophages and adipose tissue in mice*. J Biol Chem. 2012;287(41):34349–60. 10.1074/jbc.M112.410613. 22902781 10.1074/jbc.M112.410613 PMC3464541

[27] Wang Y, Jiang Q. γ-Tocotrienol inhibits lipopolysaccharide-induced interlukin-6 and granulocyte colony-stimulating factor by suppressing C/EBPβ and NF-κB in macrophages. J Nutr Biochem. 2013;24(6):1146–52. 10.1016/j.jnutbio.2012.08.015. 23246159 10.1016/j.jnutbio.2012.08.015 PMC3610800

[28] Klein S, Gastaldelli A, Yki-Järvinen H, Scherer PE. Why does obesity cause diabetes? Cell Metab. 2022;34(1):11–20. 10.1016/j.cmet.2021.12.012. 34986330 10.1016/j.cmet.2021.12.012 PMC8740746

[29] Luo H, Krigman J, Zhang R, Yang M, Sun N. Pharmacological inhibition of USP30 activates tissue-specific mitophagy. Acta Physiol (Oxf). 2021;232(3):e13666. 10.1111/apha.13666. 33890401 10.1111/apha.13666 PMC8266733

[30] Ma C, Lin Z, Yao J, Qin W, Wang X, Li Q, et al. Loss of USP10 promotes hepatocellular carcinoma proliferation by regulating the serine synthesis pathway through inhibition of LKB1 activity. Cancer Sci. 2024;115(12):3902–14. 10.1111/cas.16336. 39327097 10.1111/cas.16336 PMC11611778

[31] Purushotham A, Schug TT, Xu Q, Surapureddi S, Guo X, Li X. Hepatocyte-specific deletion of SIRT1 alters fatty acid metabolism and results in hepatic steatosis and inflammation. Cell Metab. 2009;9(4):327–38. 10.1016/j.cmet.2009.02.006. 19356714 10.1016/j.cmet.2009.02.006 PMC2668535

[32] Kundu A, Dey P, Park JH, Kim IS, Kwack SJ, Kim HS. EX-527 prevents the progression of high-fat diet-induced hepatic steatosis and fibrosis by upregulating SIRT4 in zucker rats. Cells. 2020;9(5):1101. 10.3390/cells9051101. 32365537 10.3390/cells9051101 PMC7290750

[33] Uehara K, Sostre-Colón J, Gavin M, Santoleri D, Leonard KA, Jacobs RL, et al. Activation of liver mTORC1 protects against NASH via dual regulation of VLDL-TAG secretion and de novo lipogenesis. Cell Mol Gastroenterol Hepatol. 2022;13(6):1625–47. 10.1016/j.jcmgh.2022.02.015. 35240344 10.1016/j.jcmgh.2022.02.015 PMC9046248

[34] Wang C, Yan Y, Hu L, Zhao L, Yang P, Moorhead JF, et al. Rapamycin-mediated CD36 translational suppression contributes to alleviation of hepatic steatosis. Biochem Biophys Res Commun. 2014;447(1):57–63. 10.1016/j.bbrc.2014.03.103. 24685479 10.1016/j.bbrc.2014.03.103

[35] Romacho T, Elsen M, Röhrborn D, Eckel J. Adipose tissue and its role in organ crosstalk. Acta Physiol (Oxf). 2014;210(4):733–53. 10.1111/apha.12246. 24495317 10.1111/apha.12246

[36] HematJouy S, Mohan S, Scichilone G, Mostafa A, Mahmoud AM. Adipokines in the crosstalk between adipose tissues and other organs: implications in cardiometabolic diseases. Biomedicines. 2024;12(9):2129. 10.3390/biomedicines12092129. 39335642 10.3390/biomedicines12092129 PMC11428859

[37] Azzu V, Vacca M, Virtue S, Allison M, Vidal-Puig A. Adipose tissue-liver cross talk in the control of whole-body metabolism: implications in nonalcoholic fatty liver disease. Gastroenterology. 2020;158(7):1899–912. 10.1053/j.gastro.2019.12.054. 32061598 10.1053/j.gastro.2019.12.054

[38] Zatterale F, Longo M, Naderi J, Raciti GA, Desiderio A, Miele C, et al. Chronic adipose tissue inflammation linking obesity to insulin resistance and type 2 diabetes. Front Physiol. 2019;10:1607. 10.3389/fphys.2019.01607. 32063863 10.3389/fphys.2019.01607 PMC7000657

[39] Baek JH, Kim DH, Lee J, Kim SJ, Chun KH. Galectin-1 accelerates high-fat diet-induced obesity by activation of peroxisome proliferator-activated receptor gamma (PPARγ) in mice. Cell Death Dis. 2021;12(1):66. 10.1038/s41419-020-03367-z. 33431823 10.1038/s41419-020-03367-z PMC7801586

[40] Kim MS, Kang H, Baek JH, Cho MG, Chung EJ, Kim SJ, et al. Disrupting Notch signaling related HES1 in myeloid cells reinvigorates antitumor T cell responses. Exp Hematol Oncol. 2024;13(1):122. 10.1186/s40164-024-00588-2. 39702544 10.1186/s40164-024-00588-2 PMC11660887

[41] Bakiallah AE, Damayanti DS, Nogueira R, Choi HS, Chun KH. LPS stimulation-induced regulation of LECT2 expression via TLR4 in hepatocytes. BMB Rep. 2025;58(6):250–6. 10.5483/BMBRep.2025-0046. 40495481 10.5483/BMBRep.2025-0046 PMC12207439

[42] Jang JH, Jung J, Kang HG, Kim W, Kim WJ, Lee H, et al. Kindlin-1 promotes gastric cancer cell motility through the Wnt/β-catenin signaling pathway. Sci Rep. 2025;15(1):2481. 10.1038/s41598-025-86220-7. 39833319 10.1038/s41598-025-86220-7 PMC11756408

