# Supplementary material for: Pharmacological inhibition of Ubiquitin-Specific Peptidase 10 (USP10) with spautin-1 attenuates adipogenesis through CCAAT/Enhancer-Binding Protein Beta (C/EBPβ) destabilization
Source: Mol Biomed. 2025 Dec 19;6:142. doi: 10.1186/s43556-025-00389-x (PMC12717316; doi:10.1186/s43556-025-00389-x)

**Pharmacological Inhibition of Ubiquitin-Specific Peptidase 10 (USP10) with Spautin-1 Attenuates Adipogenesis through CCAAT/Enhancer-Binding Protein beta (C/EBPβ) Destabilization**

Zolzaya Erdenebileg^1, 2^, Desy Damayanti Simamora^1, 2^, Joong-Kwon Park^1, 2^, Rosana Nogueira^1, 2^, Young Bin Kim^1^, Jeong-Yun Choi^1^, Hyeon-Gu Kang^1, 2^, Hack Sun Choi^1, 2^, Jung-Hwan Baek^3^, Kyung-Hee Chun^1, 2, 3, 4, *^

^1^Department of Biochemistry & Molecular Biology, Yonsei University College of Medicine, Republic of Korea

^2^School of Medical Science, Brain Korea 21 Project, Yonsei University College of Medicine, Republic of Korea

^3^JLBiotherapeutics, Republic of Korea

^4^Affiliate Faculty, Pohang University of Science and Technology, Republic of Korea

*Corresponding author: Kyung-Hee Chun

Department of Biochemistry & Molecular Biology, Yonsei University College of Medicine,

50-1 Yonsei-ro, Seodaemun-gu, Seoul 03722, Republic of Korea

Tel: 82-10-7666-6291, email: khchun@yuhs.ac

ORCID: 0000-0002-9867-7321

**Supplementary Figures and legends**

**
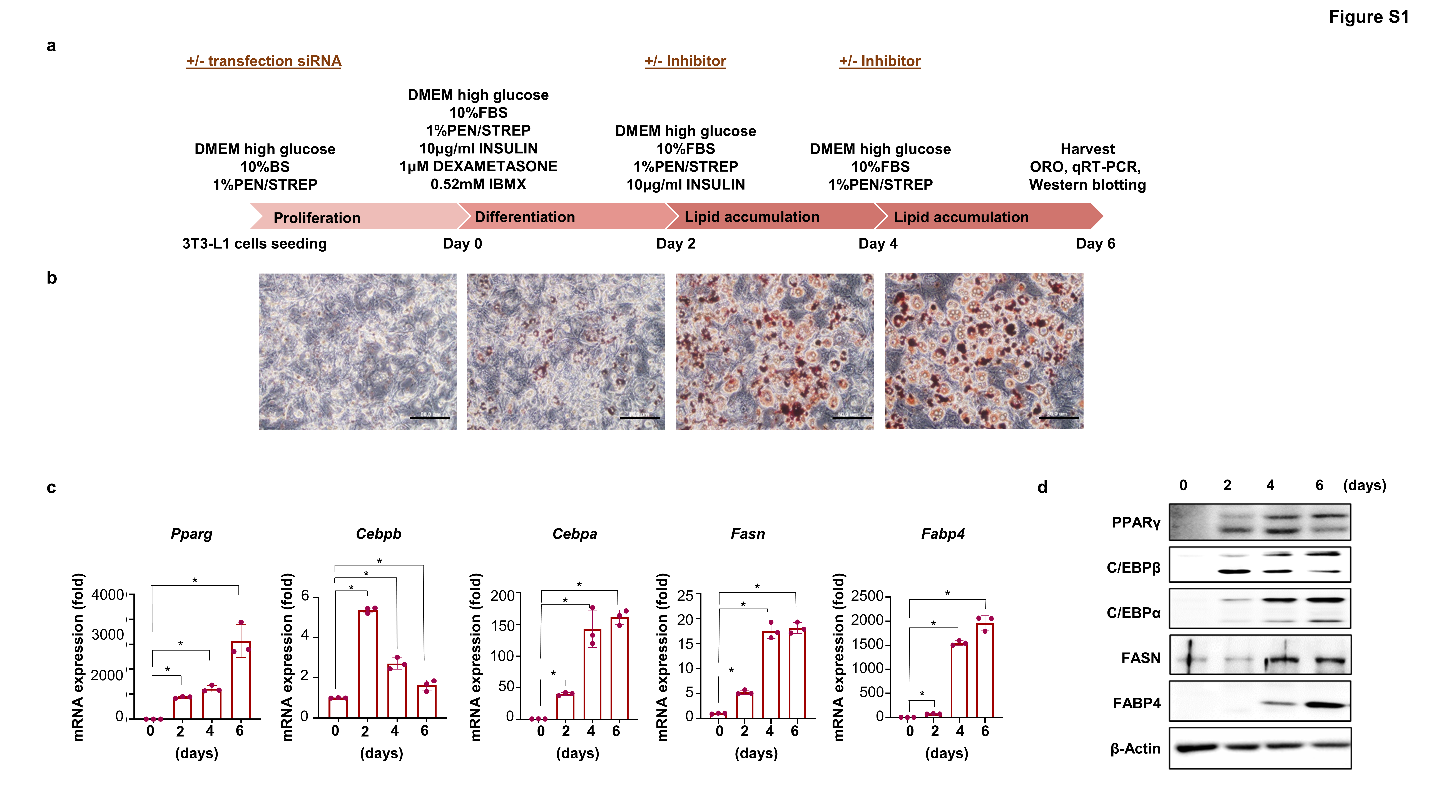
**

**Figure S1**. **Representation of study design and phenotypic characteristics of 3T3-L1 cells.** All phenotypes were observed in 3T3-L1 cells. (a) Study design of the pre-mature adipocyte to mature adipocyte and schedule of inhibitor treatment or siRNA transfection. (b) Microscopic images of post Oil Red O staining on different days of differentiation. (c-d) mRNA and protein expression levels of adipogenic factors during differentiation on different days. Data are mean ± SD from n = 3 independent experiments (RT–qPCR in technical triplicates). Statistics: one-way ANOVA with Dunnett’s post hoc versus vehicle for multi-dose comparisons; where applicable, two-tailed unpaired *t*-test. Significance: *p < 0.05.


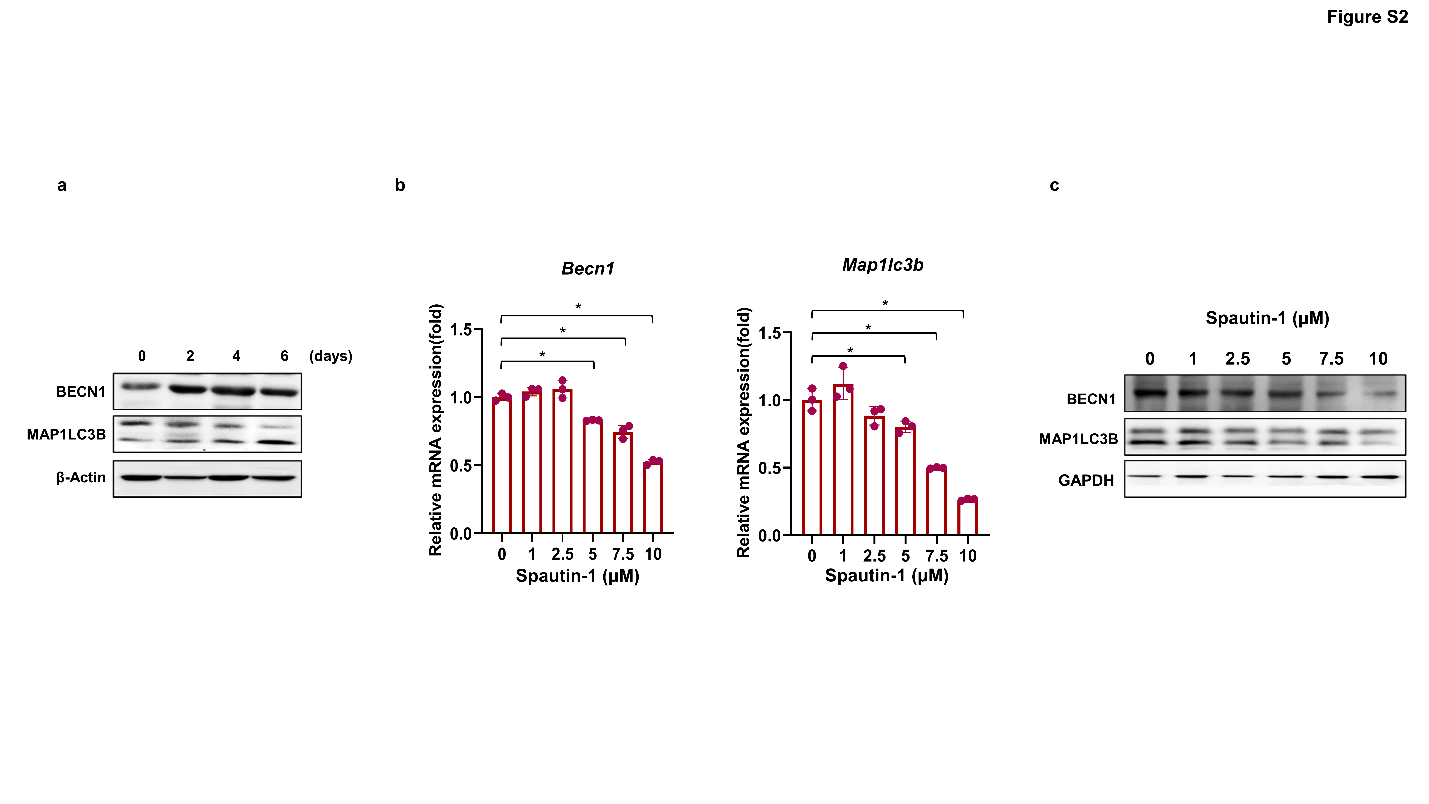


**Figure S2**. **Spautin-1 suppresses autophagy markers during adipogenesis in 3T3-L1 cells.** (a) BECN1 and MAP1LC3B increase during early adipogenic induction in 3T3-L1 cells, it was detected by western blotting. (b-c) Spautin-1 treatment significantly reduces BECN1 and MAP1LC3B at the protein and mRNA levels Data are mean ± SD from n = 3 independent experiments (RT–qPCR in technical triplicates). Statistics: one-way ANOVA with Dunnett’s post hoc versus vehicle for multi-dose comparisons; where applicable, two-tailed unpaired *t*-test. Significance: *p < 0.05.


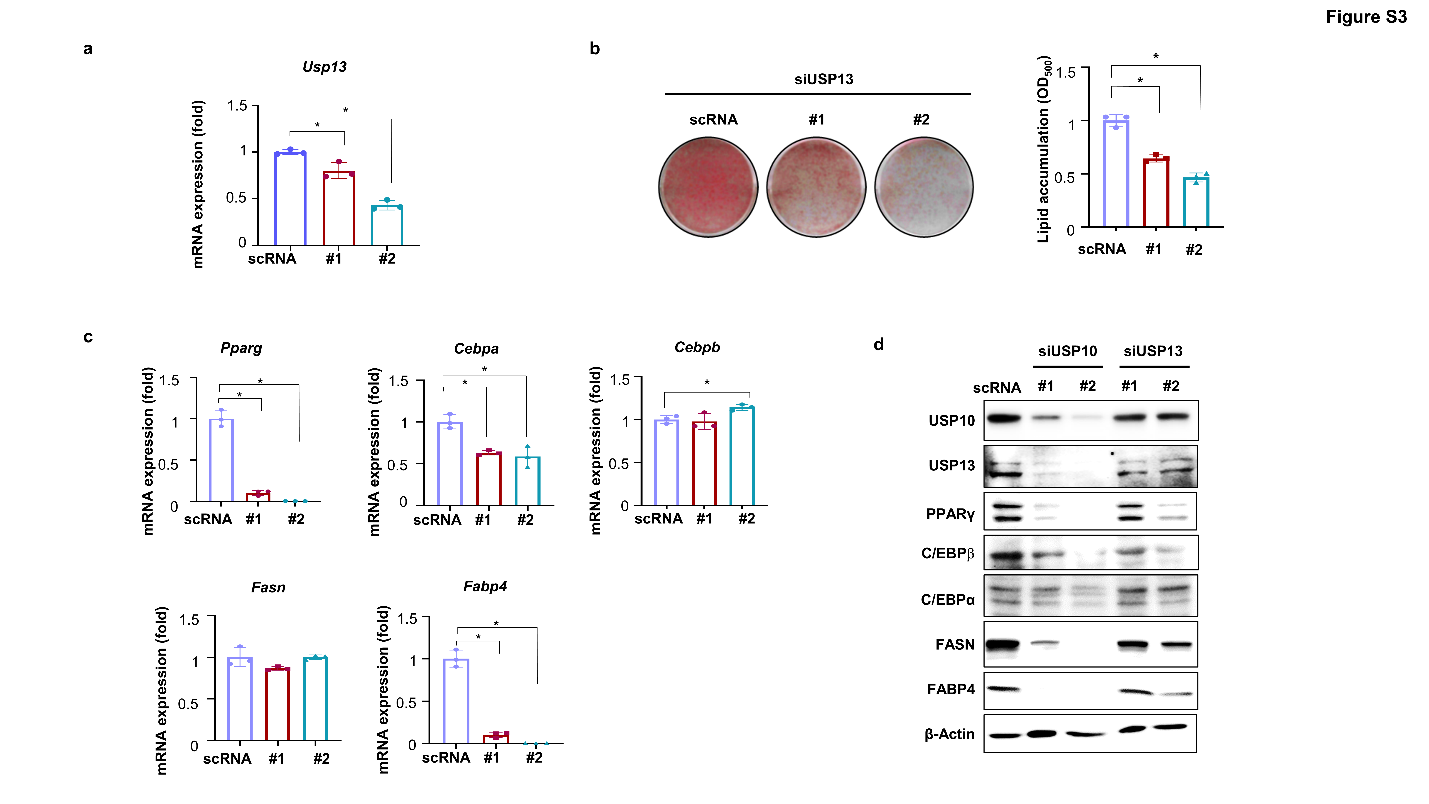


**Figure S3**. **Effects of USP13 knockdown on adipocyte differentiation in 3T3-L1 cells.** (a) USP13 siRNA knockdown efficiency mRNA of *Usp13* was measured by qRT-PCR. (b) Oil Red O staining at full differentiation with quantification. (c) mRNA levels of *Pparg, Cebpa, Cebpb, Fasn,* and *Fabp4* (qRT-PCR, normalized to Gapdh). (d) Protein levels of PPARγ, C/EBPα, C/EBPβ, FASN, and FABP4 (immunoblot; β-Actin loading control). Data are mean ± SD from n = 3 independent experiments (RT–qPCR in technical triplicates). Statistics: one-way ANOVA with Dunnett’s post hoc versus vehicle for multi-dose comparisons; where applicable, two-tailed unpaired *t*-test. Significance: *p < 0.05.


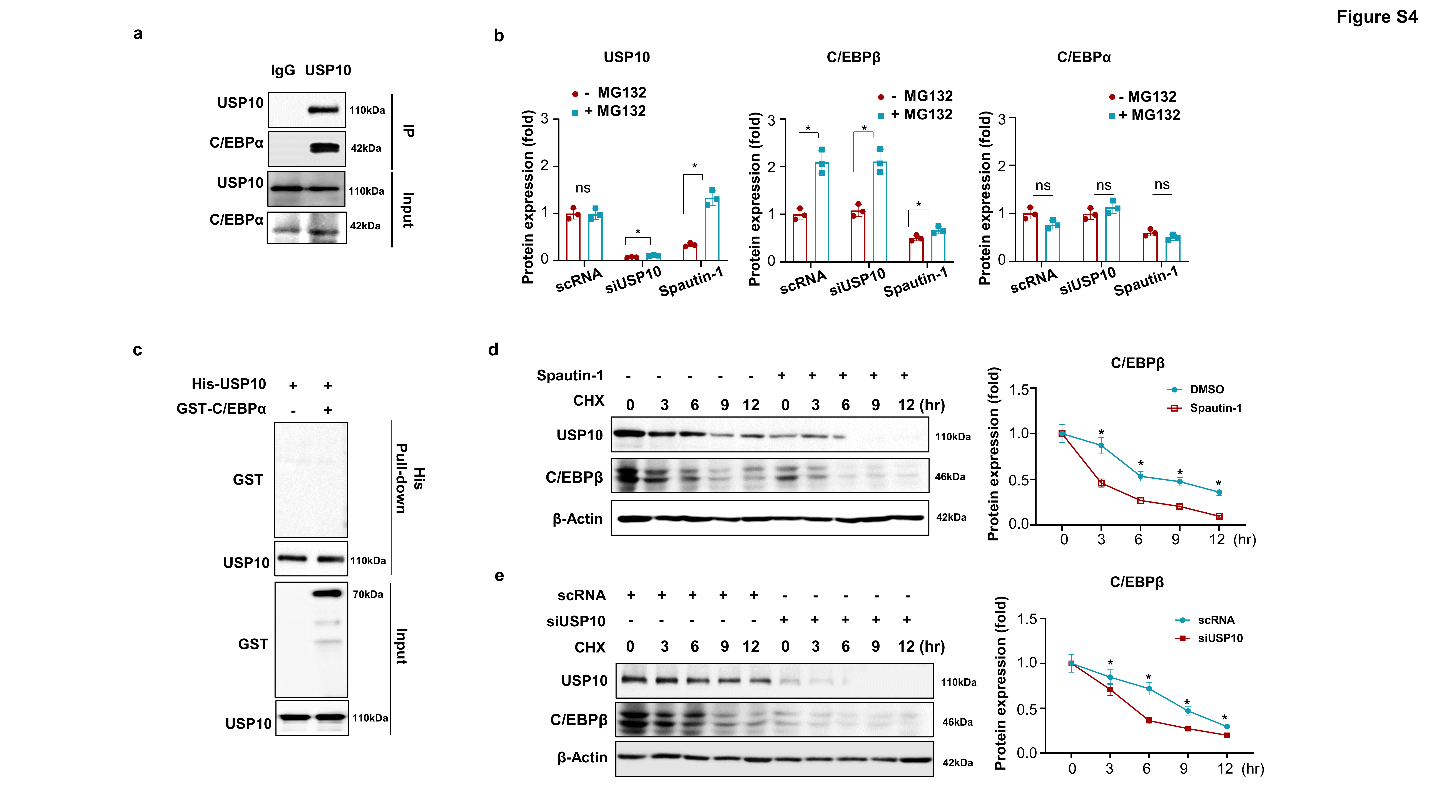


**Figure S4**. **USP10 interacts with C/EBPβ and regulates its protein stability.** All phenotypes were observed in HEK-293 cells. (a) Endogenous immunoprecipitation (IP) of USP10 with C/EBPα (b) Relative protein levels of USP10, C/EBPβ, and C/EBPα. were normalized using β-Actin (See Figure 4b for blots) (c) His-tag pull-down assays demonstrating a direct interaction between USP10 and C/EBPβ (Figure 4c) but not between USP10 and C/EBPα, separately. (d-e) Protein half-life of C/EBPβ through USP10 inhibition with spautin-1 treatment or siRNA against USP10. Cells were treated with spautin-1 for 24 h and transfected with Usp10 siRNA for 48 h. Relative protein levels of C/EBPβ were normalized using β-Actin. Data represent the mean ± SD (n = 3 for each sample) p < 0.05 for DMSO vs. spautin-1 and scRNA vs. siUSP10. Statistical significance was identified using an unpaired two-tailed *t*-test. Significance: *p < 0.05.


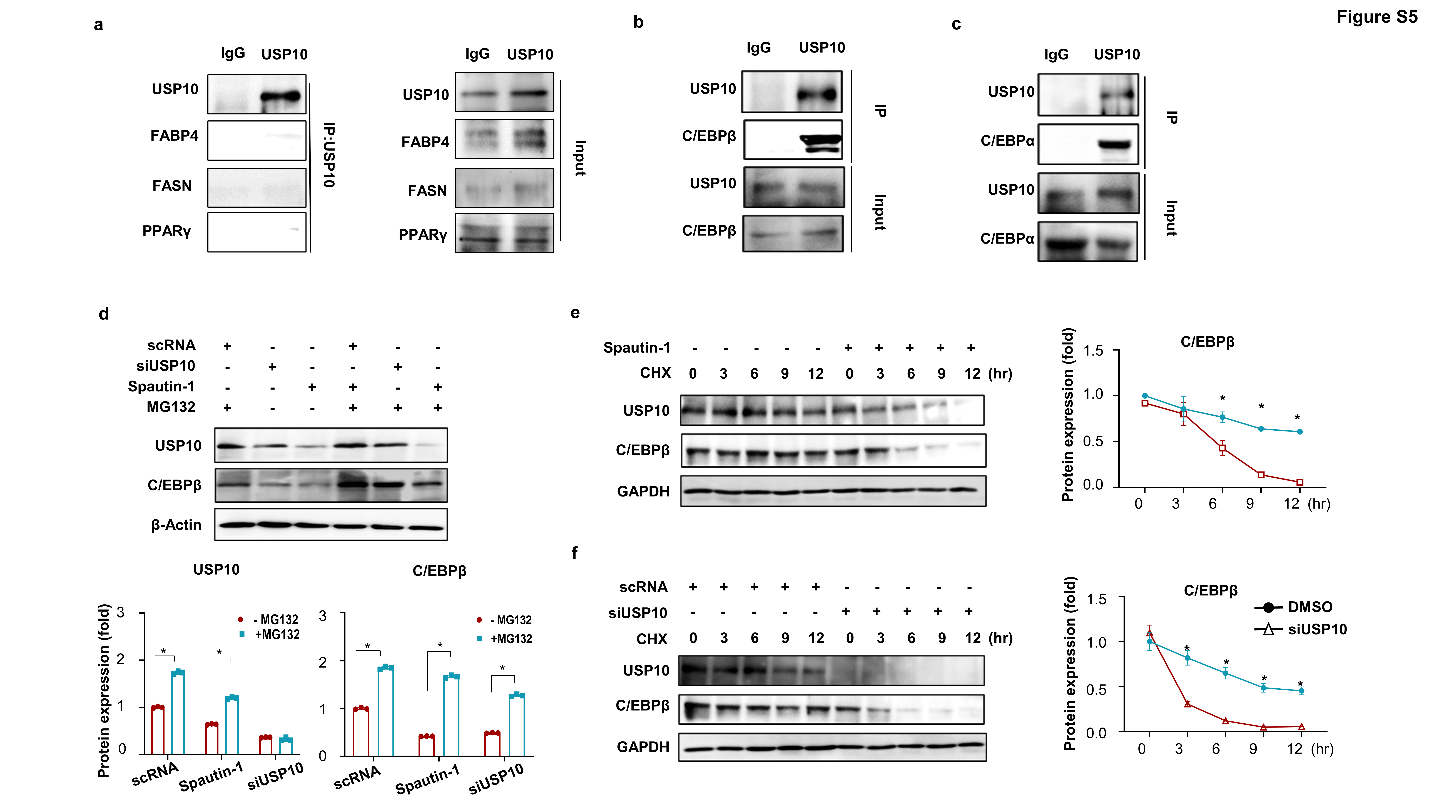


**Figure S5**. **USP10 interacts with C/EBPβ and regulates its protein stability.** All phenotypes were observed in 3T3-L1 cells. (a) Endogenous immunoprecipitation (IP) of USP10 with adipogenic/lipogenic factors in adipocytes. 3T3-L1 adipocyte lysates were immunoprecipitated with control IgG or anti-USP10 and immunoblotted (IB) for USP10, PPARγ, FASN, and FABP4. Inputs (5–10%) are shown; PPARγ, FASN and FABP4 are enriched in the USP10 IP relative to IgG. β-Actin served as an input loading control. Representative of ≥2 independent experiments. (b-c) Interaction between endogenous USP10 and C/EBPβ or USP10 and C/EBPα was confirmed separately. Cells were immunoprecipitated using IgG and USP10 antibodies (d). Effect of Usp10 knockdown and spautin-1 on the protein stability of C/EBPβ. 3T3-L1 cells were transfected with siRNA against USP10 or treated with spautin-1 for 48 h. Relative protein levels of USP10 and C/EBPβ were normalized using β-Actin (on the bottom). (e-f) Protein half-life of C/EBPβ through USP10 inhibition with spautin-1 treatment or siRNA against USP10. Cells were treated with spautin-1 for 24 h and transfected with USP10 siRNA for 48 h. Relative protein levels of C/EBPβ were normalized using β-Actin. Data represent the mean ± SD (n = 3 for each sample), *p < 0.05 for DMSO vs. spautin-1 and scRNA vs. siUsp10. Statistical significance was identified using an unpaired two-tailed t-test.


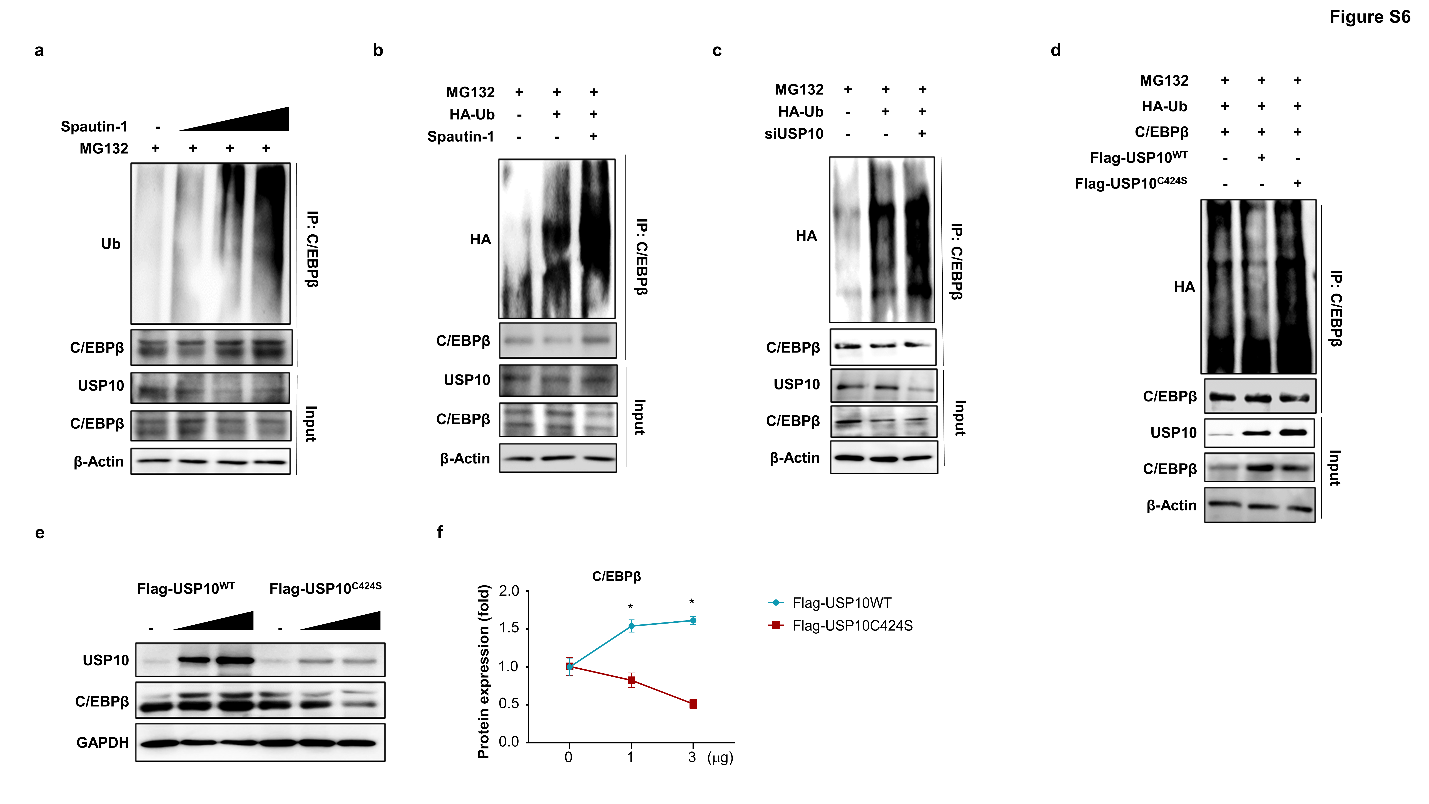


**Figure S6**. **USP10 regulates C/EBPβ protein stability through deubiquitination.** All phenotypes were observed on 3T3-L1 cells through a Western blotting assay. (a-b) Spautin-1 treatment promoting C/EBPβ ubiquitination dose-dependently. Cells were treated with spautin-1 for 24 h, followed by 10 μM of MG132 for 8 h. (c) USP10 knockdown inducing C/EBPβ ubiquitination. Cells were transfected with siRNA against Usp10 and treated with 10 μM of MG132 for 8 h before the experiments. (d) USP10 catalytic activity regulates C/EBPβ ubiquitination. Transfection with either the USP10 WT or C424S mutant cells was followed by 10 μM of MG132 for 8 h. (e-f) USP10 WT and C242S mutant forms regulate C/EBPβ protein level dose-dependently. We used TransIT X2 transfection delivery system (MIR 6004, Mirus Bio, WI, USA) followed by manufacturer’s protocol. Cells were transiently transfected with USP10 WT and C242S mutant. Relative protein levels of C/EBPβ were normalized using GAPDH for comparison. Data represent the mean ± SD (n = 3 for each sample) for WT vs. C424S. Statistical significance was identified using a two-tailed unpaired t-test. Significance: *p < 0.05.


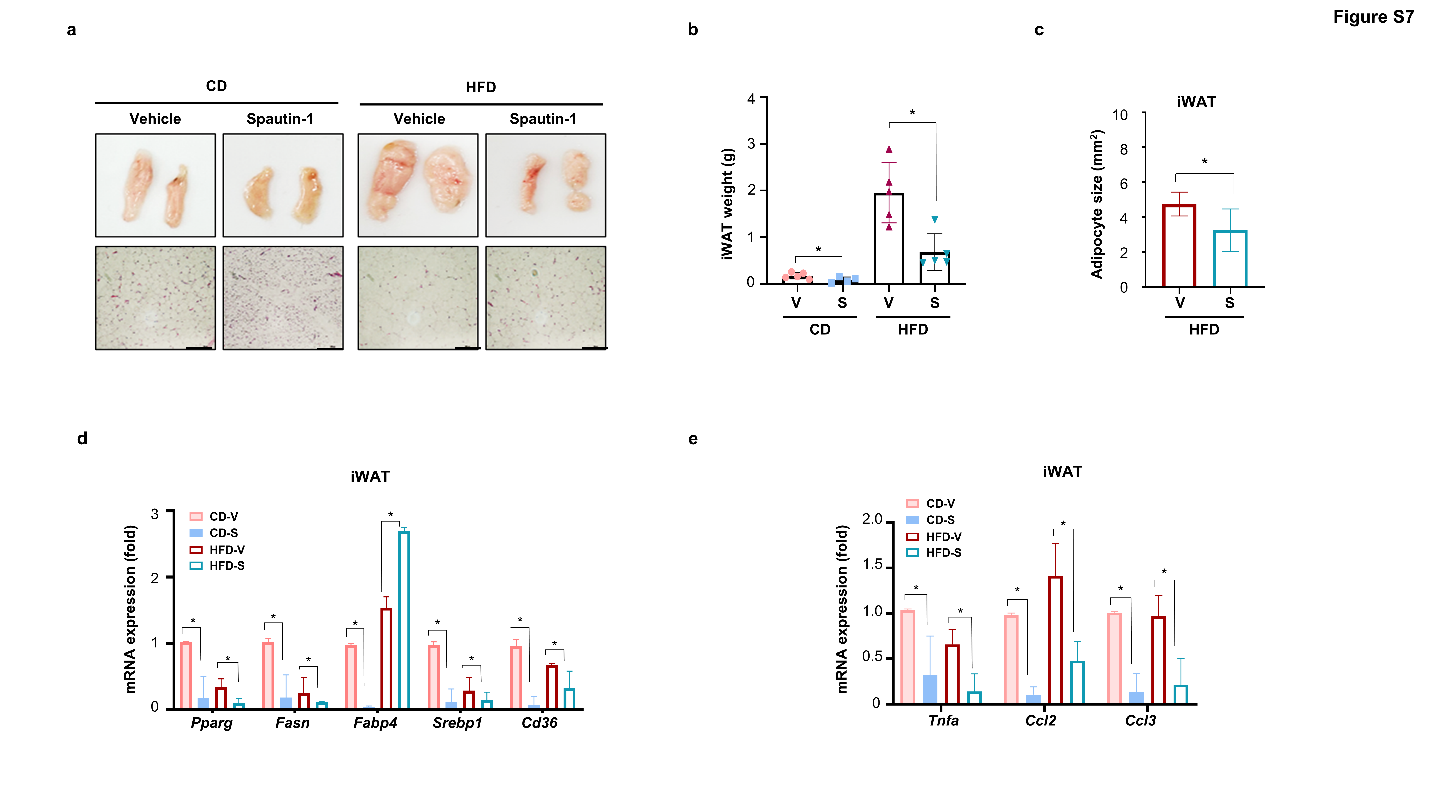


**Figure S7**. **Spautin-1 reduces adiposity, adipocyte size, and pro-adipogenic/pro-inflammatory programs in iWAT.** All endpoints were assessed after 14 weeks on chow diet (CD) or high-fat diet (HFD). (a) Representative gross images of iWAT pads and H&E-stained sections (scale bar, 100μm). (b) iWAT weight at endpoint (per pad). (c) Adipocyte area quantification from H&E sections using ImageJ/Fiji (Adiposoft/fixed-threshold workflow); ≥300 adipocytes per mouse, blinded analysis; per-mouse means plotted. (d) RT–qPCR of adipogenic/lipogenic transcripts in iWAT *(Pparg, Fasn, Fabp4, Srebp1,* and *Cd36*); β-Actin as reference; data expressed relative to vehicle within diet. (e) RT–qPCR of inflammatory genes in iWAT *(Tnfa, Ccl2,* and *Ccl3*); β-Actin as reference. Data are mean ± SD; n = 5 mice/group. Statistics: within-diet comparisons by two-tailed unpaired *t-*test (or one-way ANOVA with Dunnett’s post hoc test where applicable). Significance: *p < 0.05 versus vehicle within the same diet.

**Supplementary Tables**


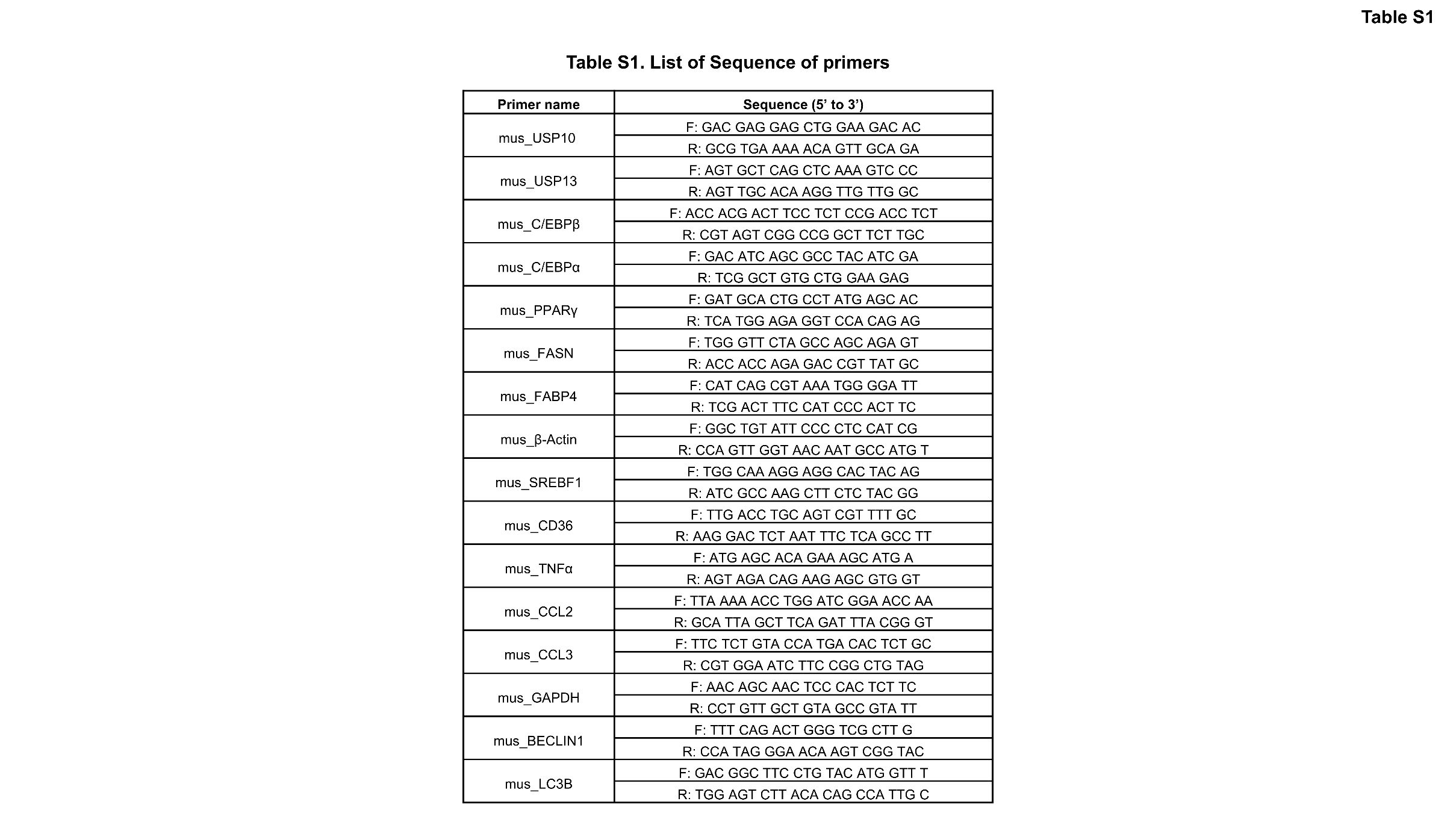


**Table S1. List of Sequence of primers**

**
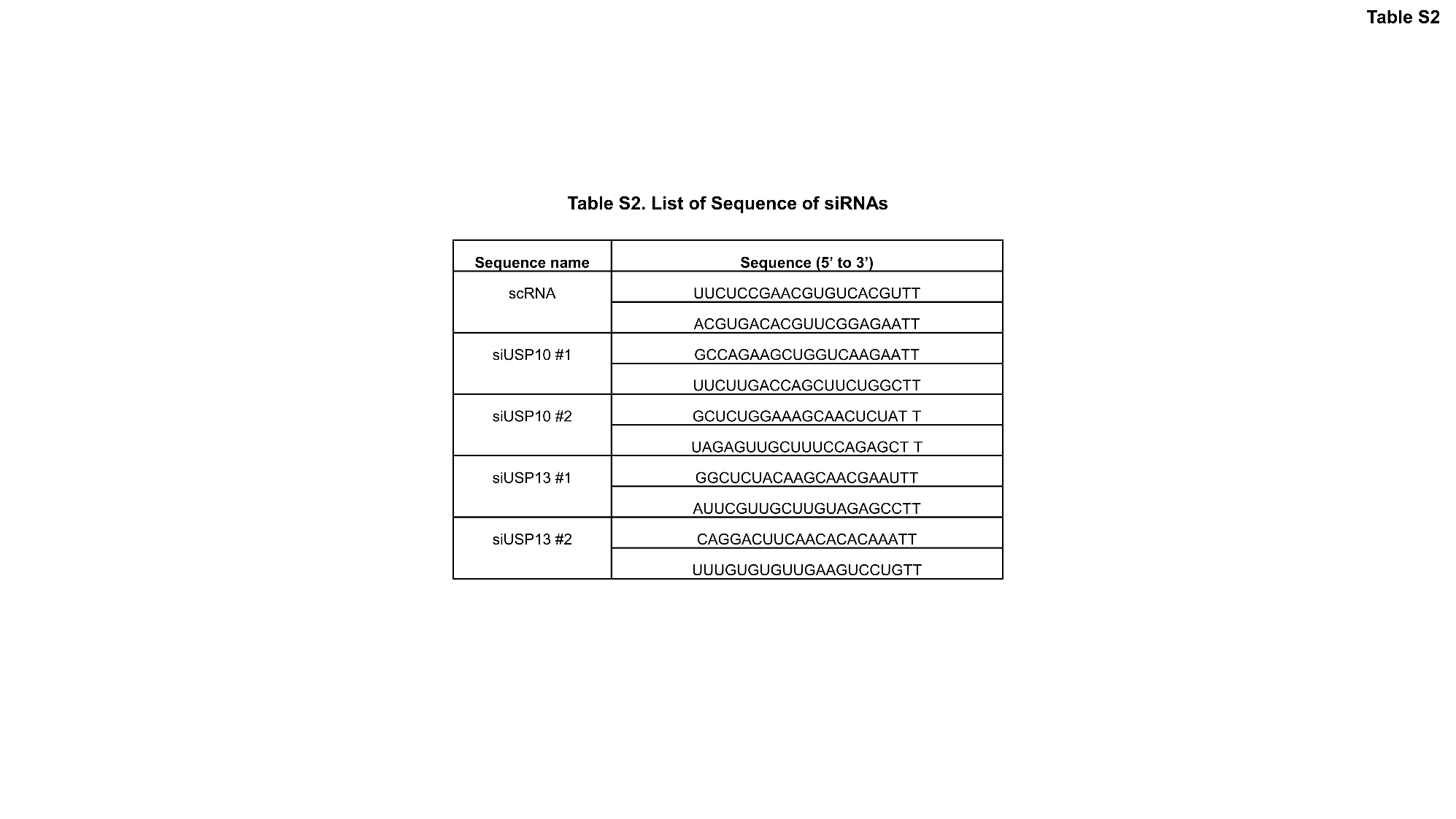
 Table S2. List of Sequence of siRNAs**


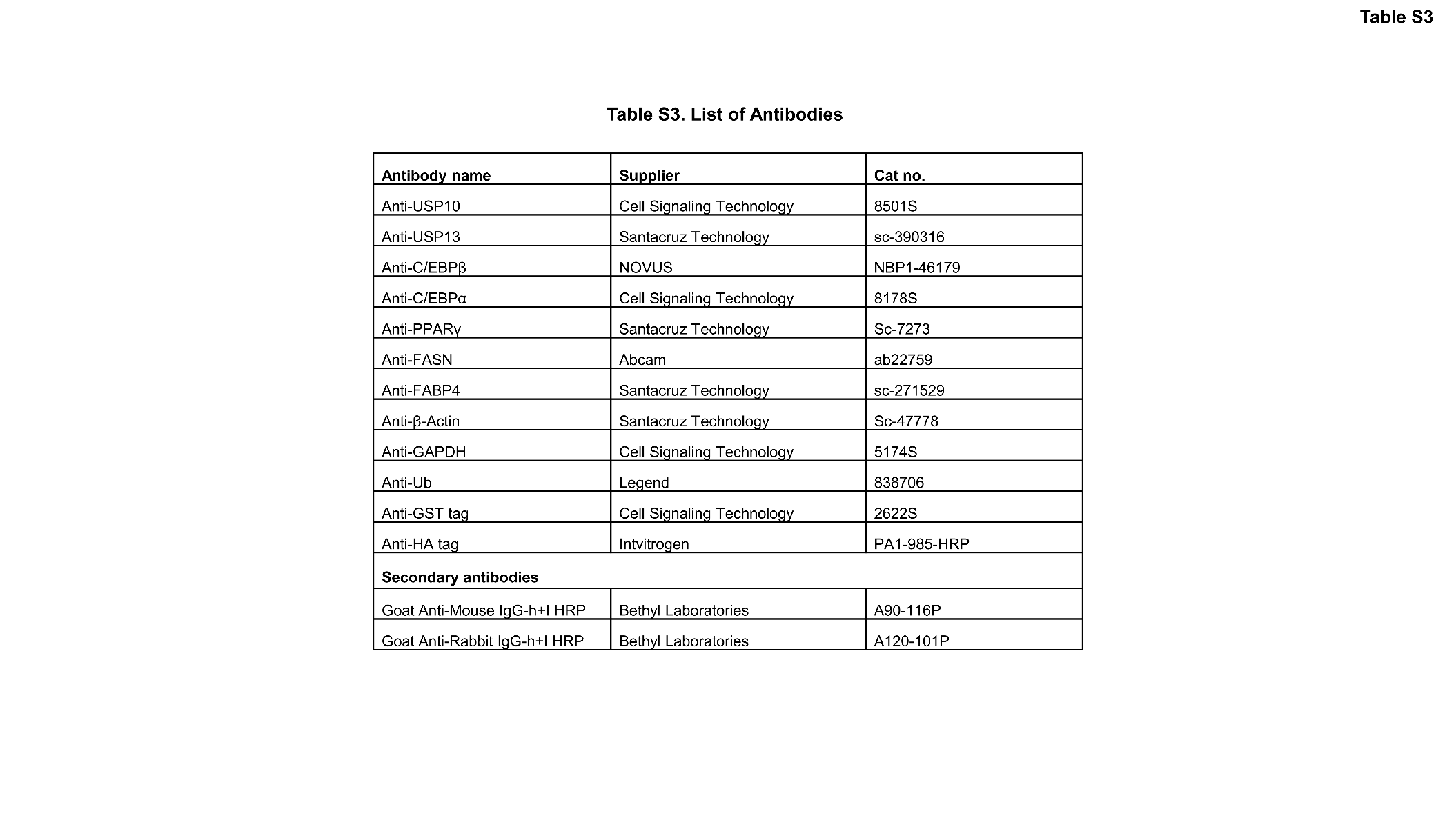


**Table S3. List of Antibodies**

**Original Blots**

**
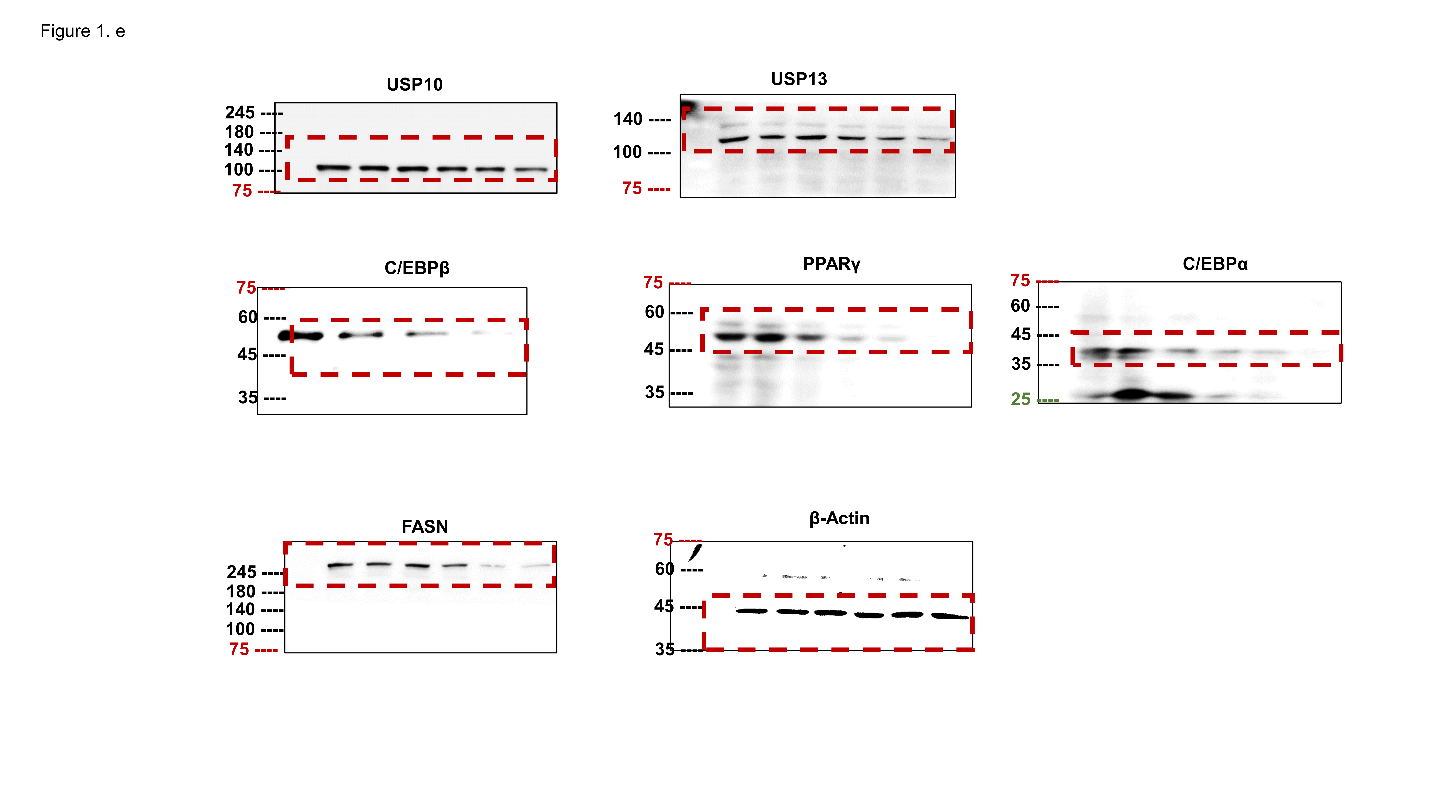

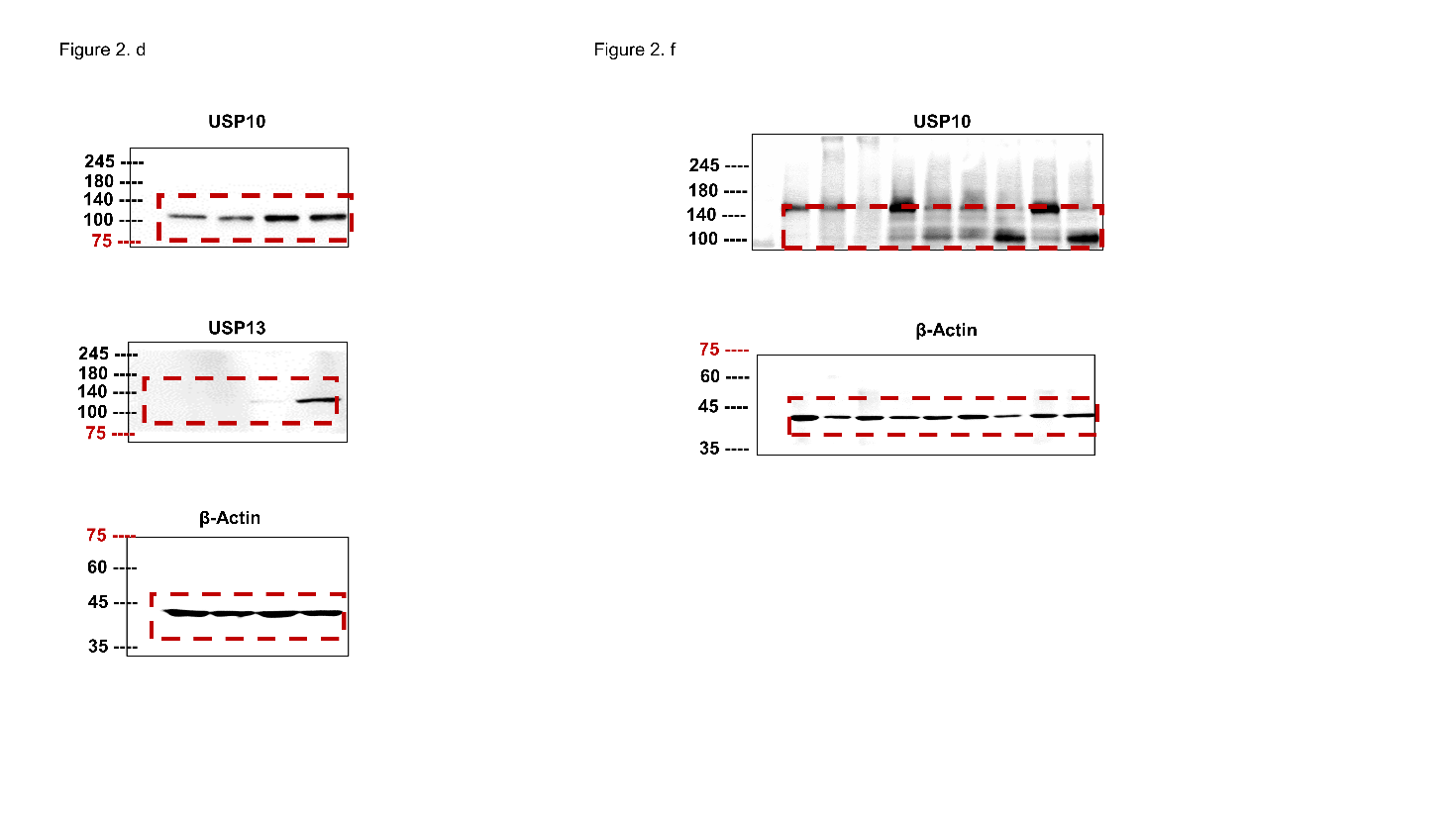
**


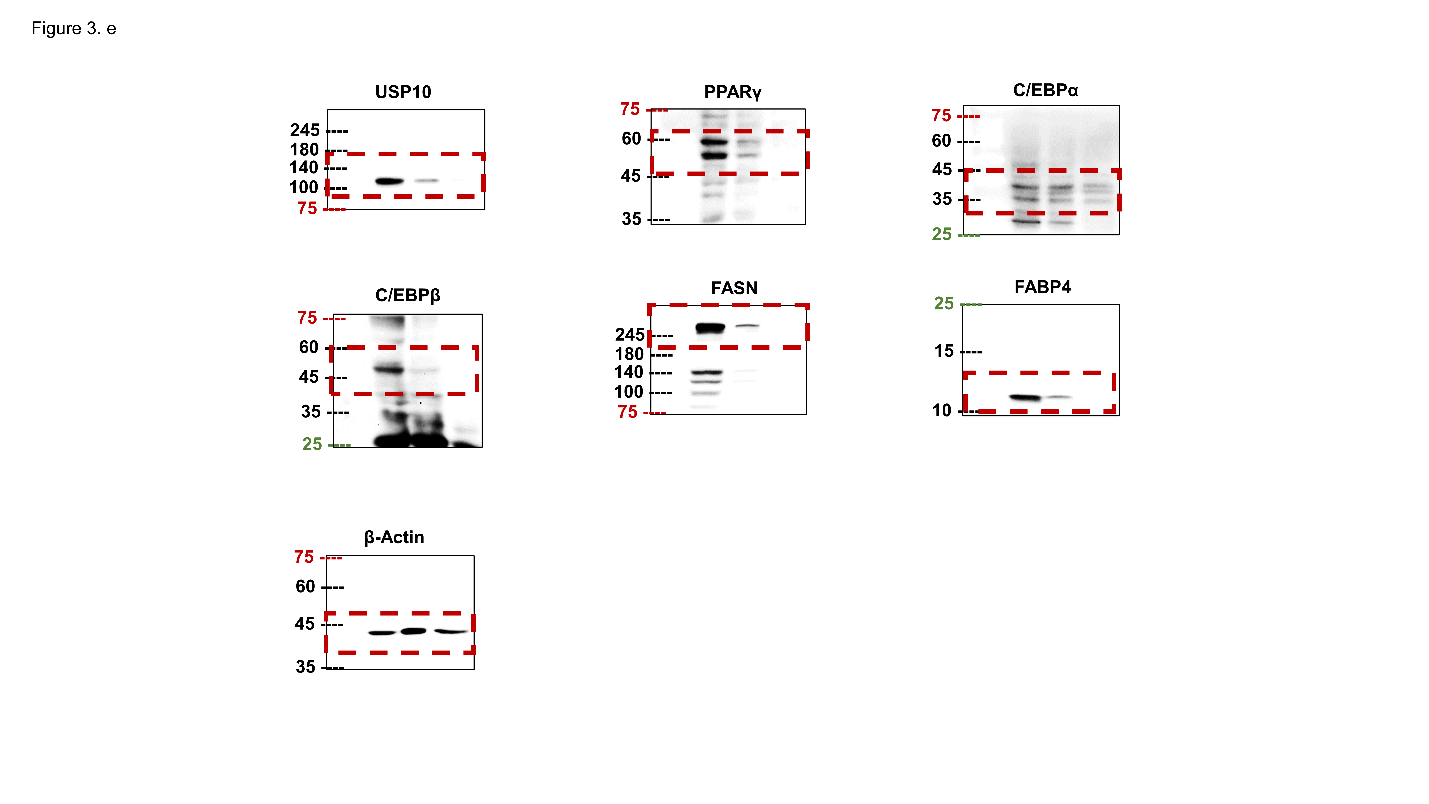

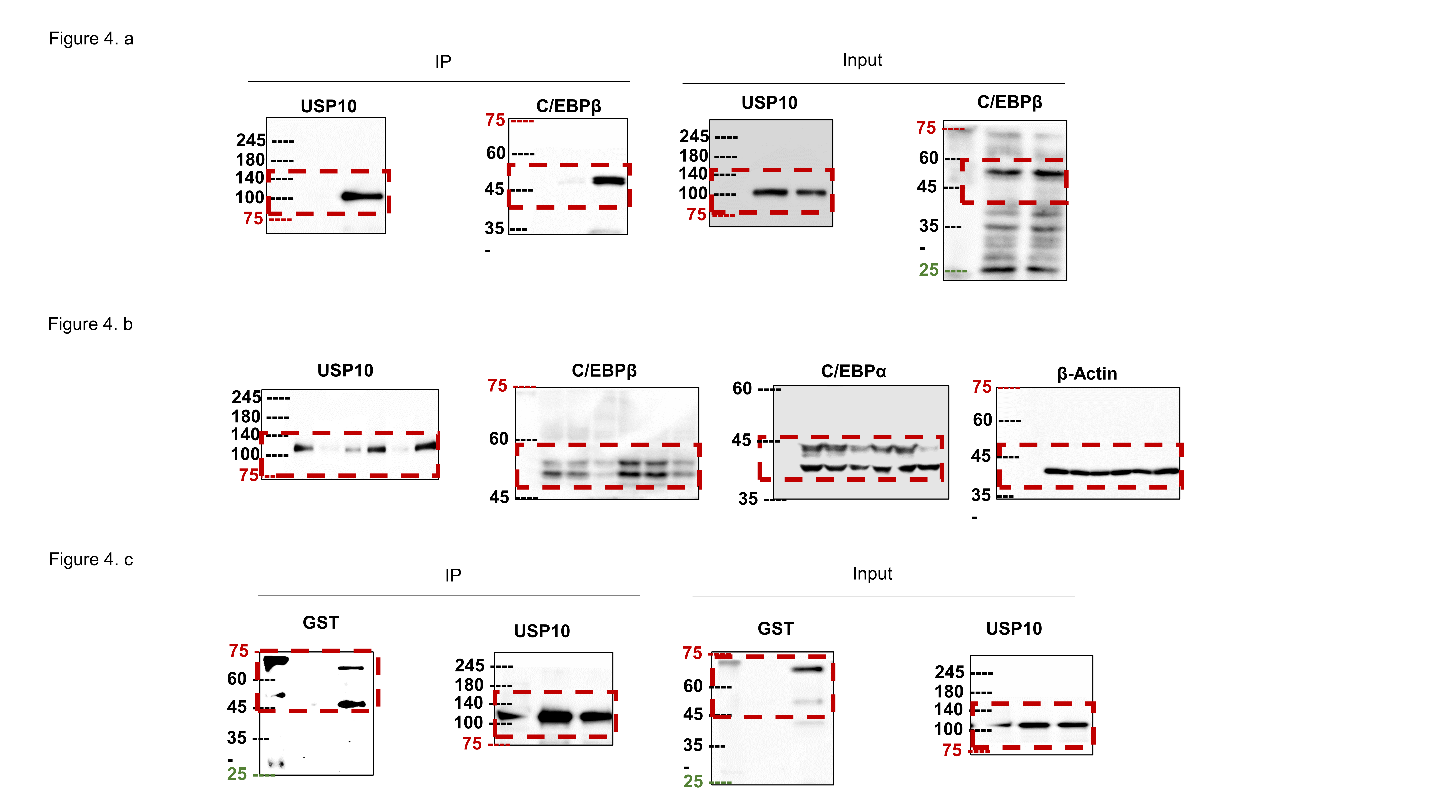


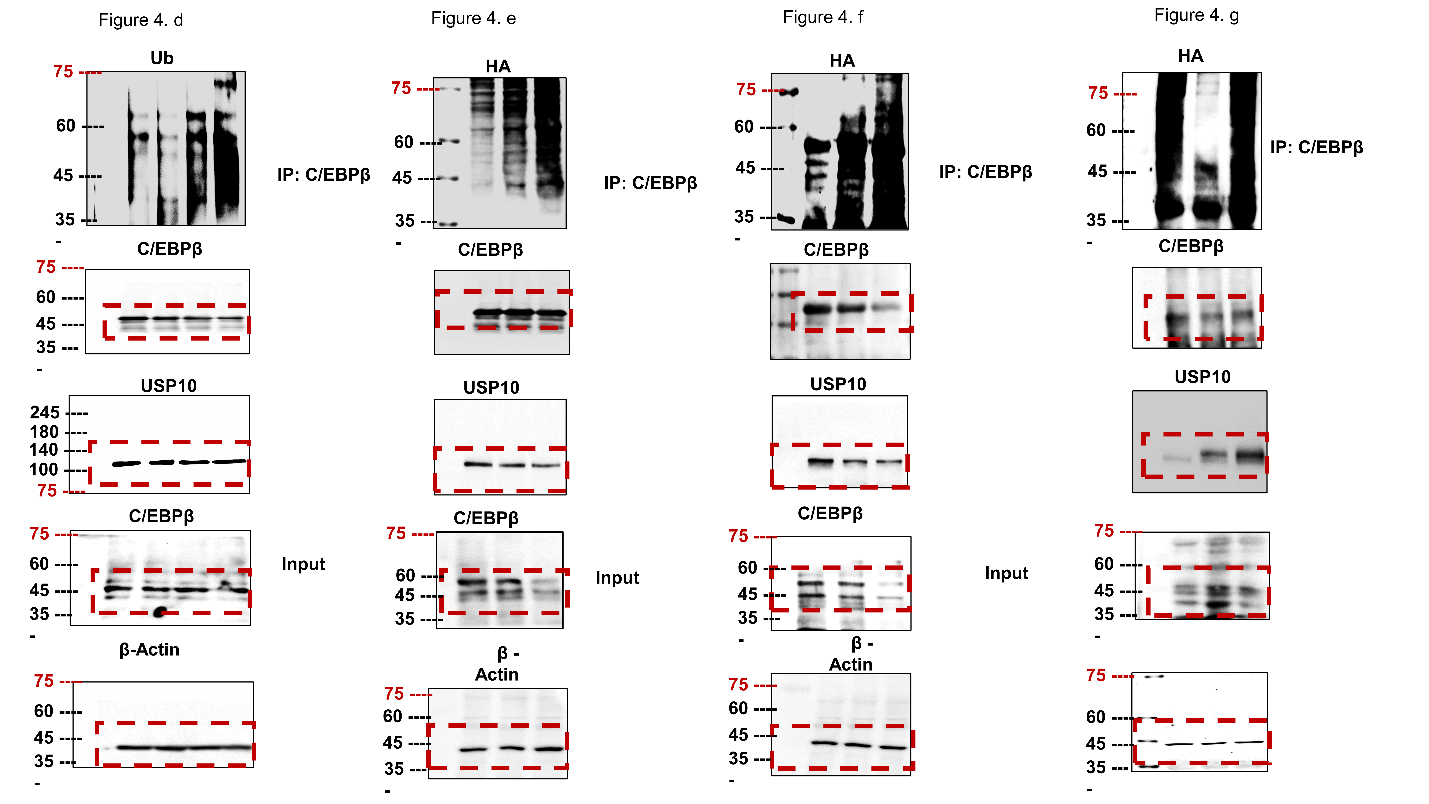


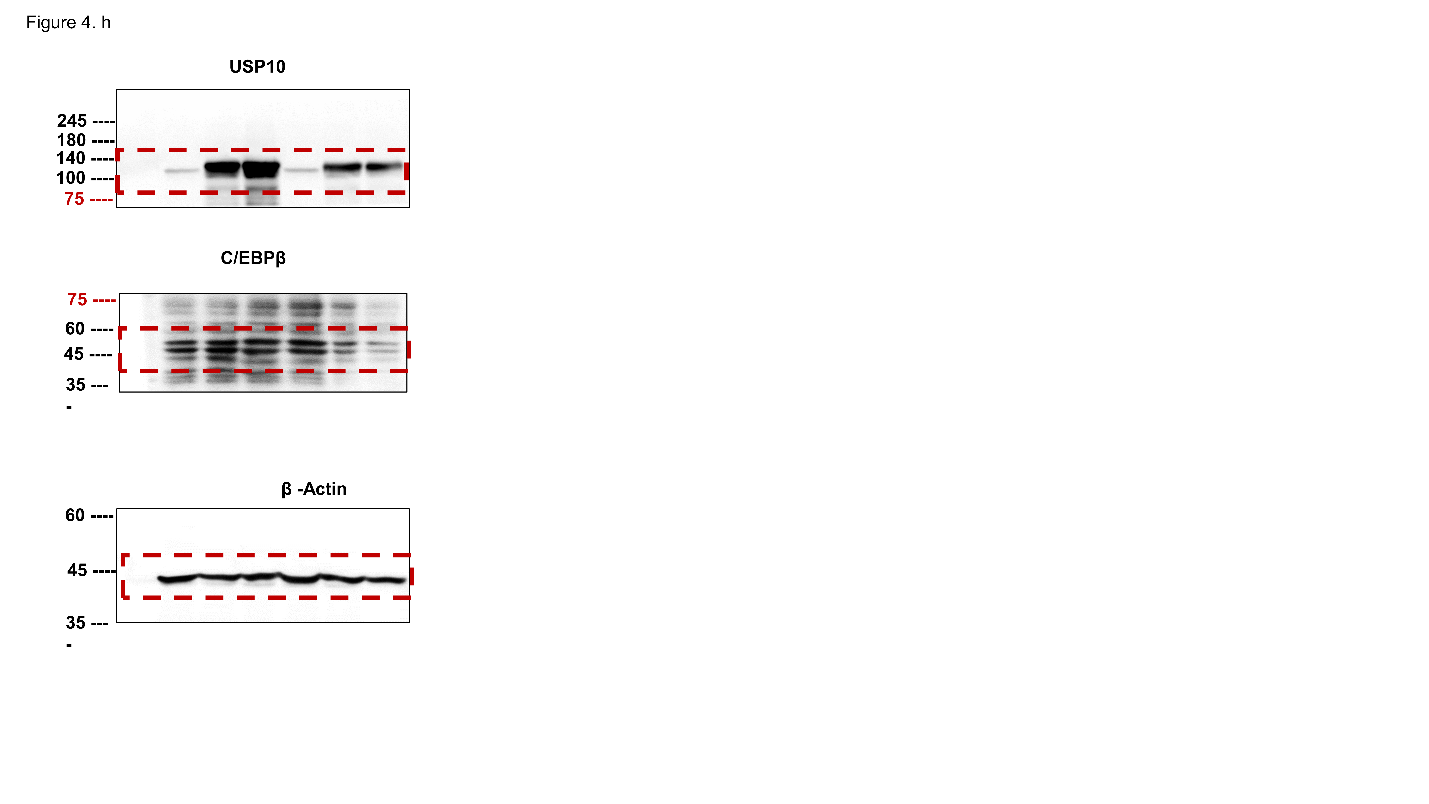


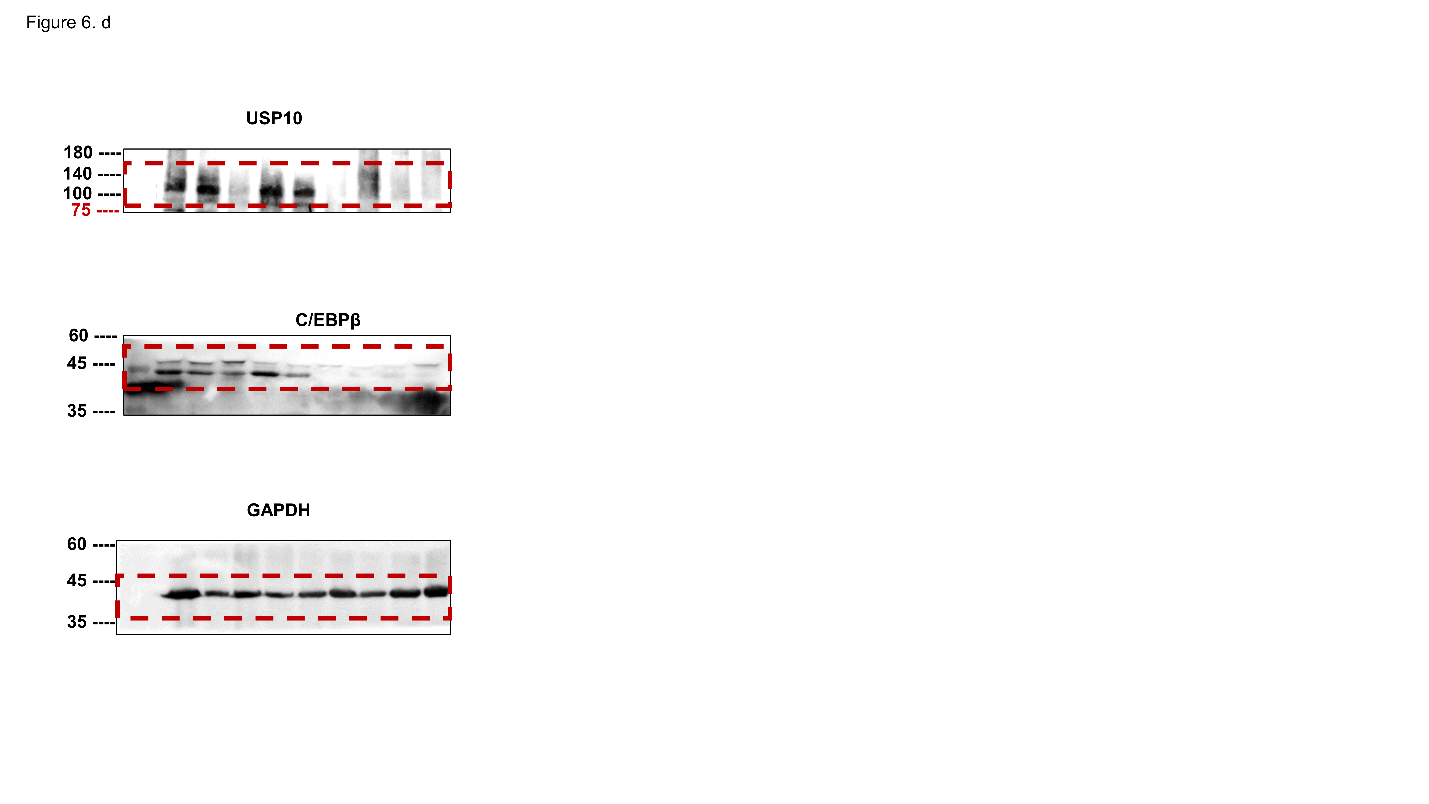

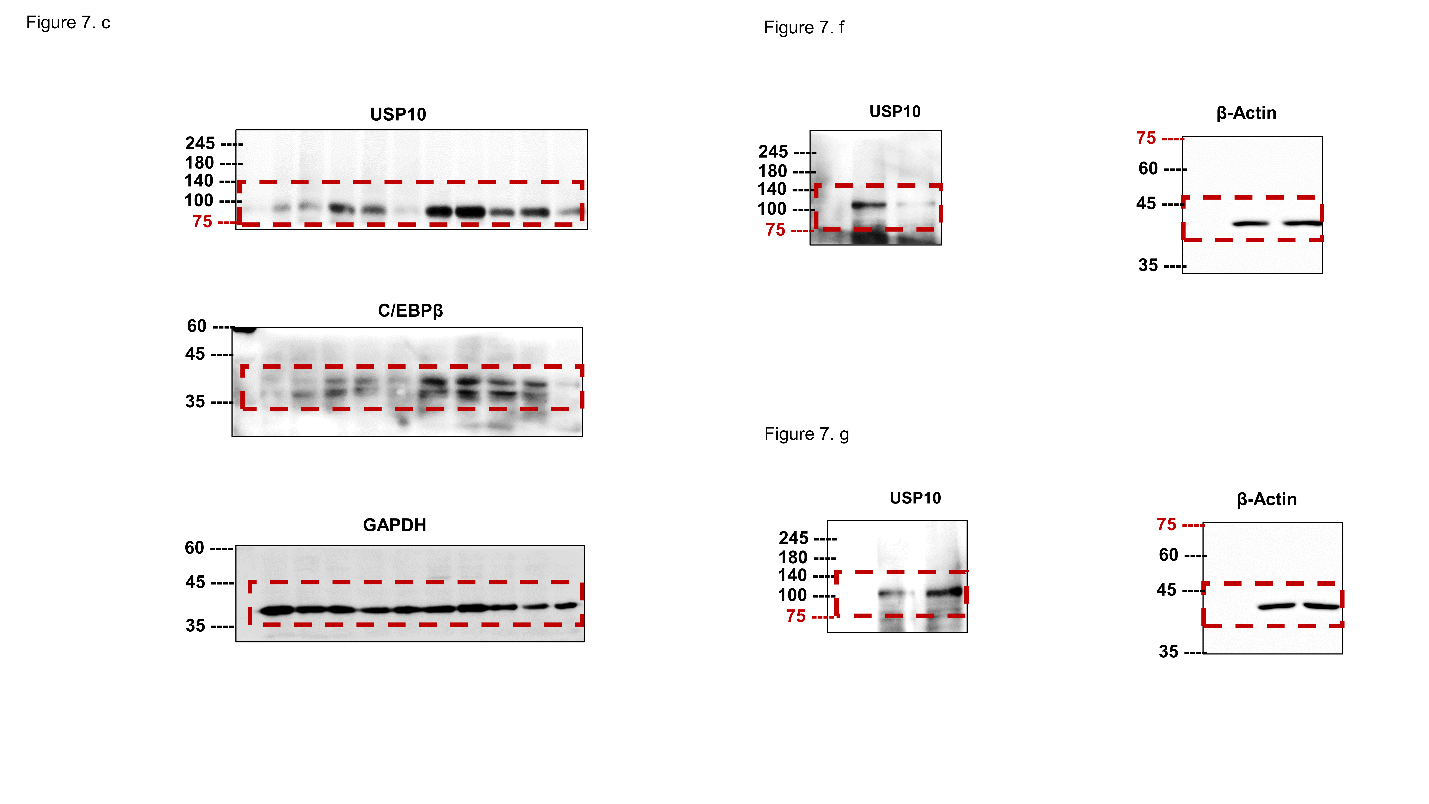


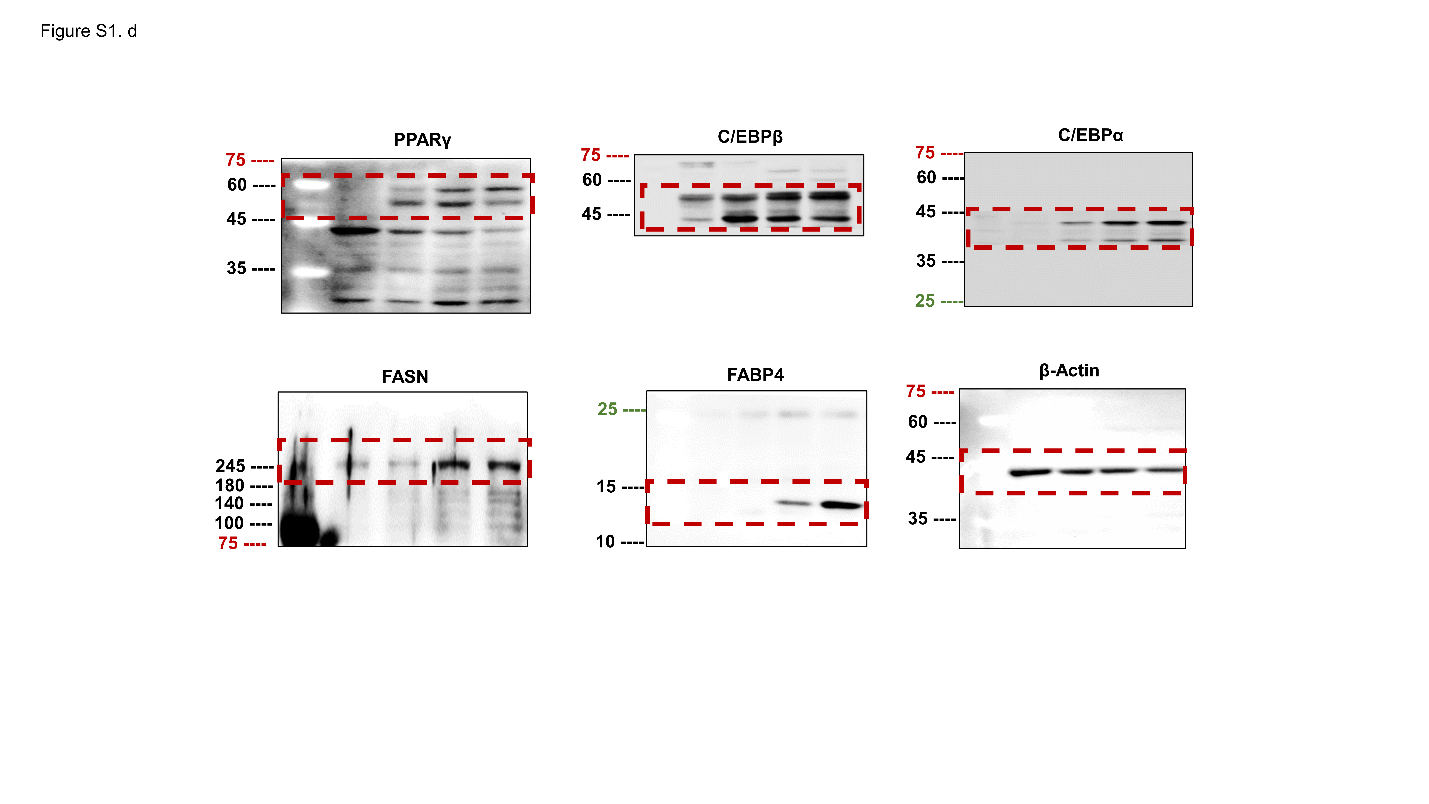


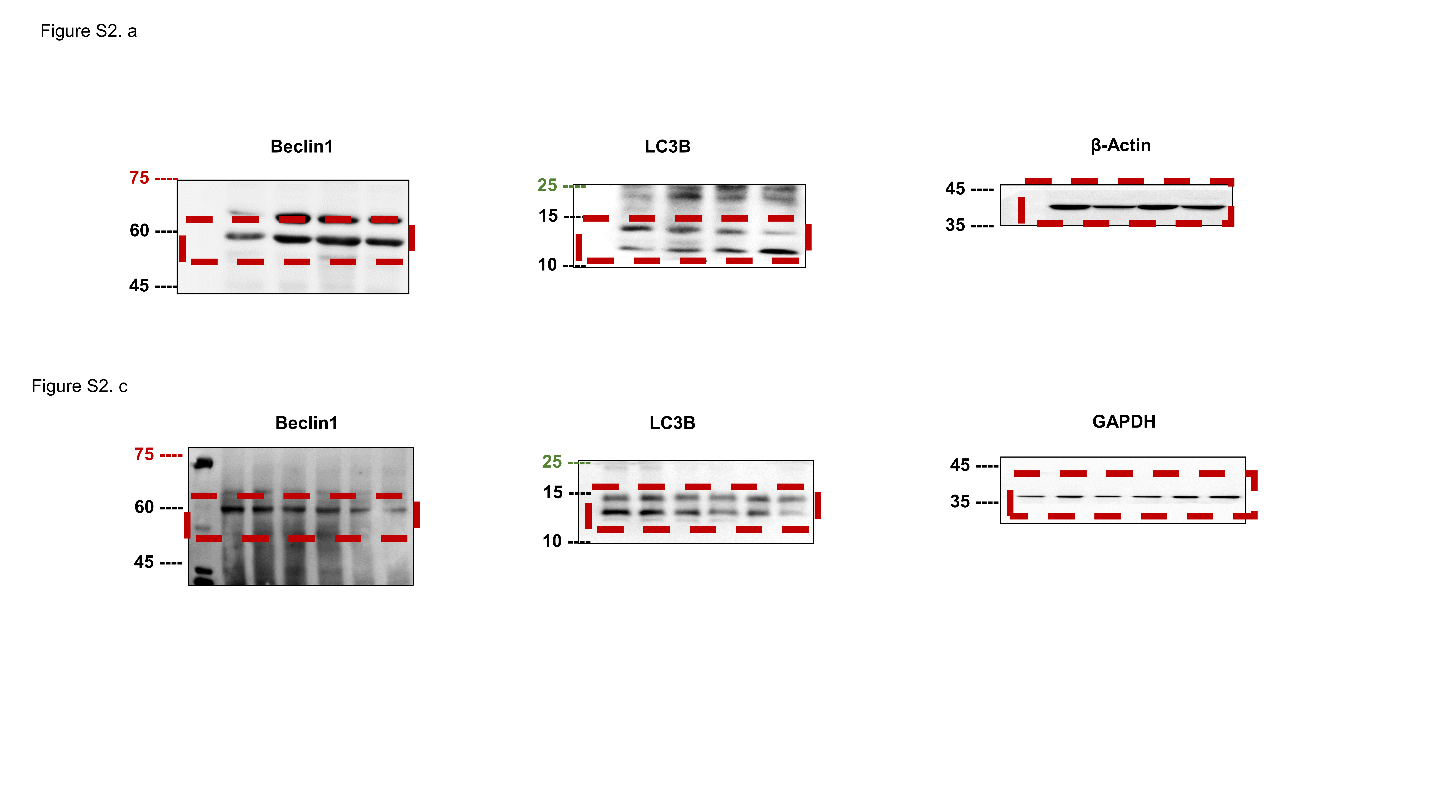


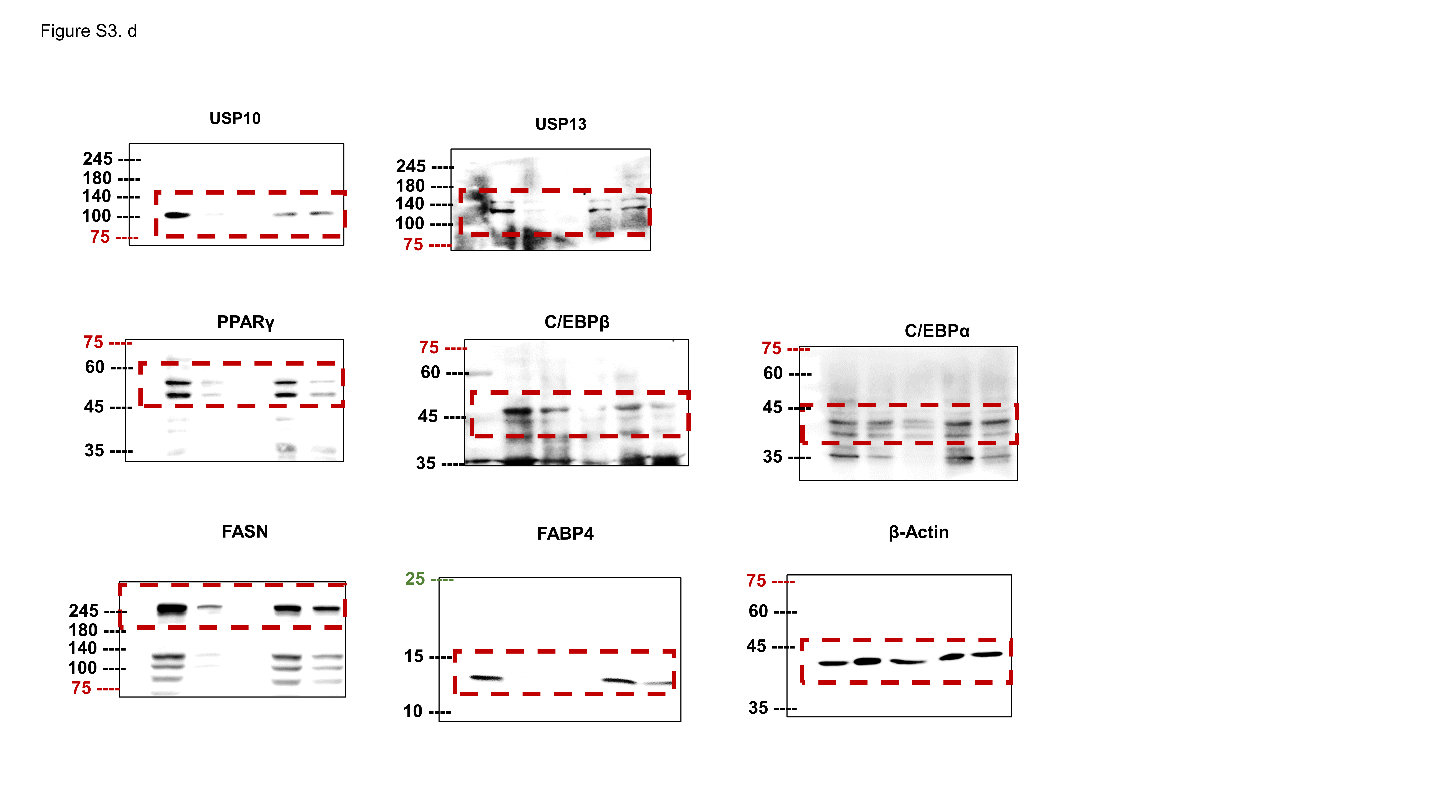


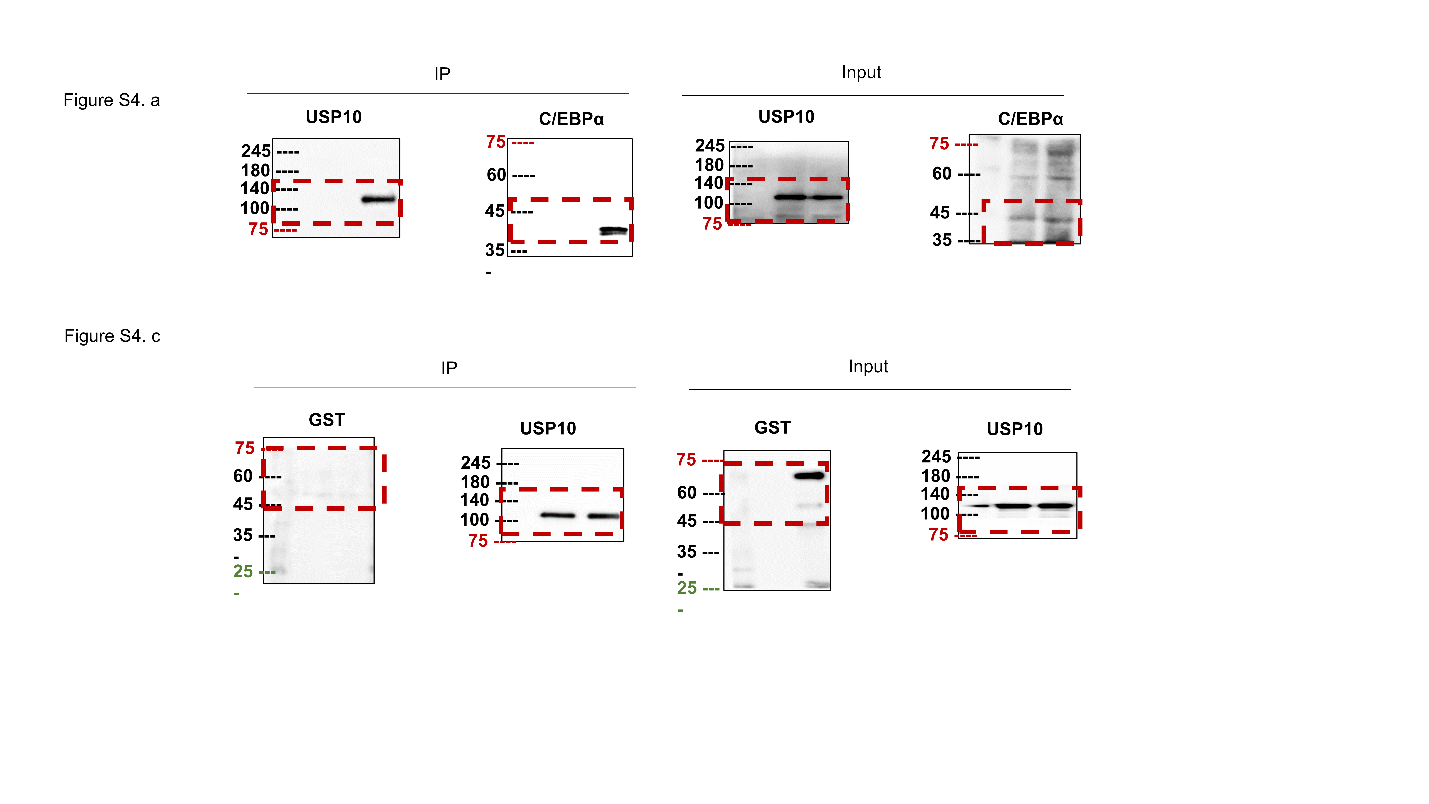


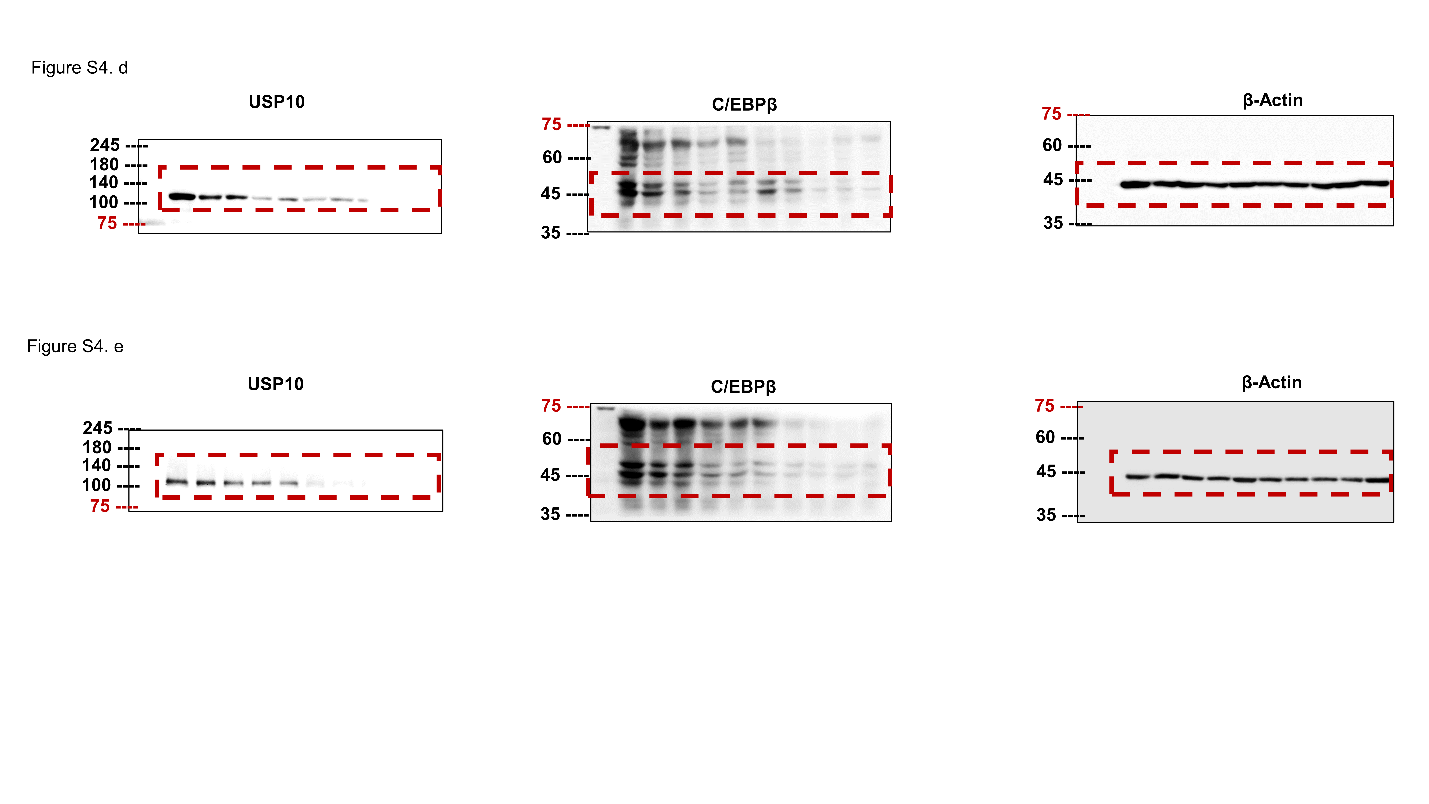


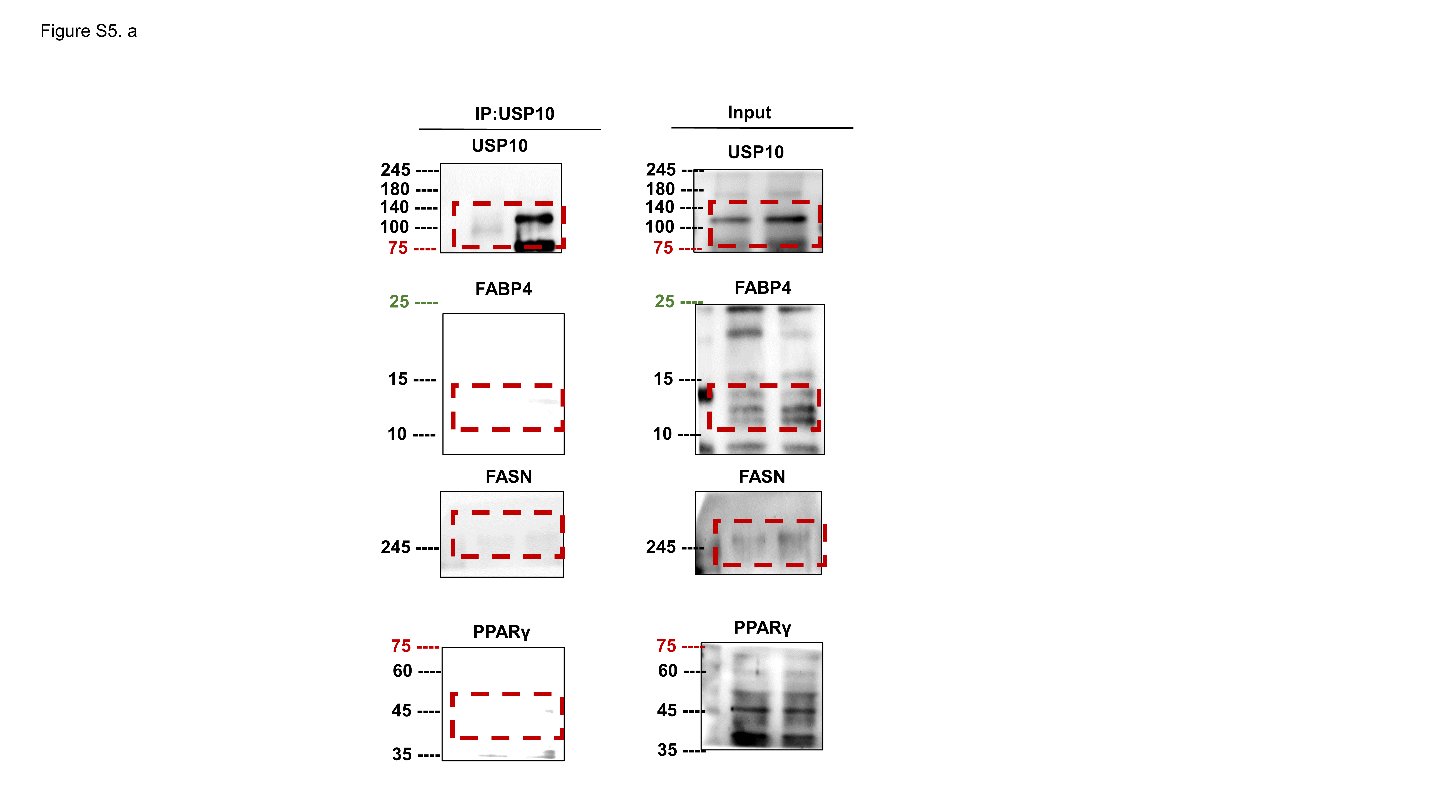


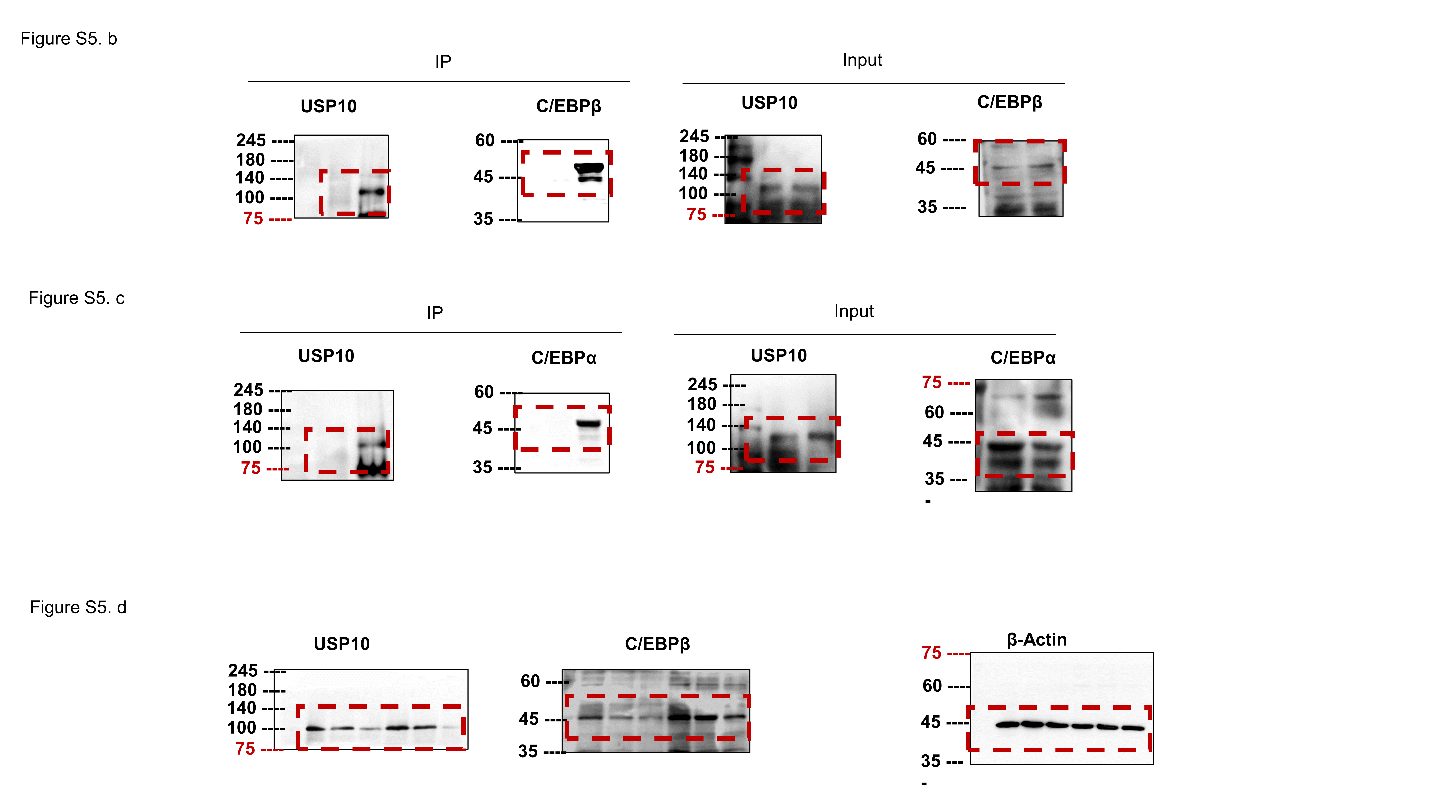


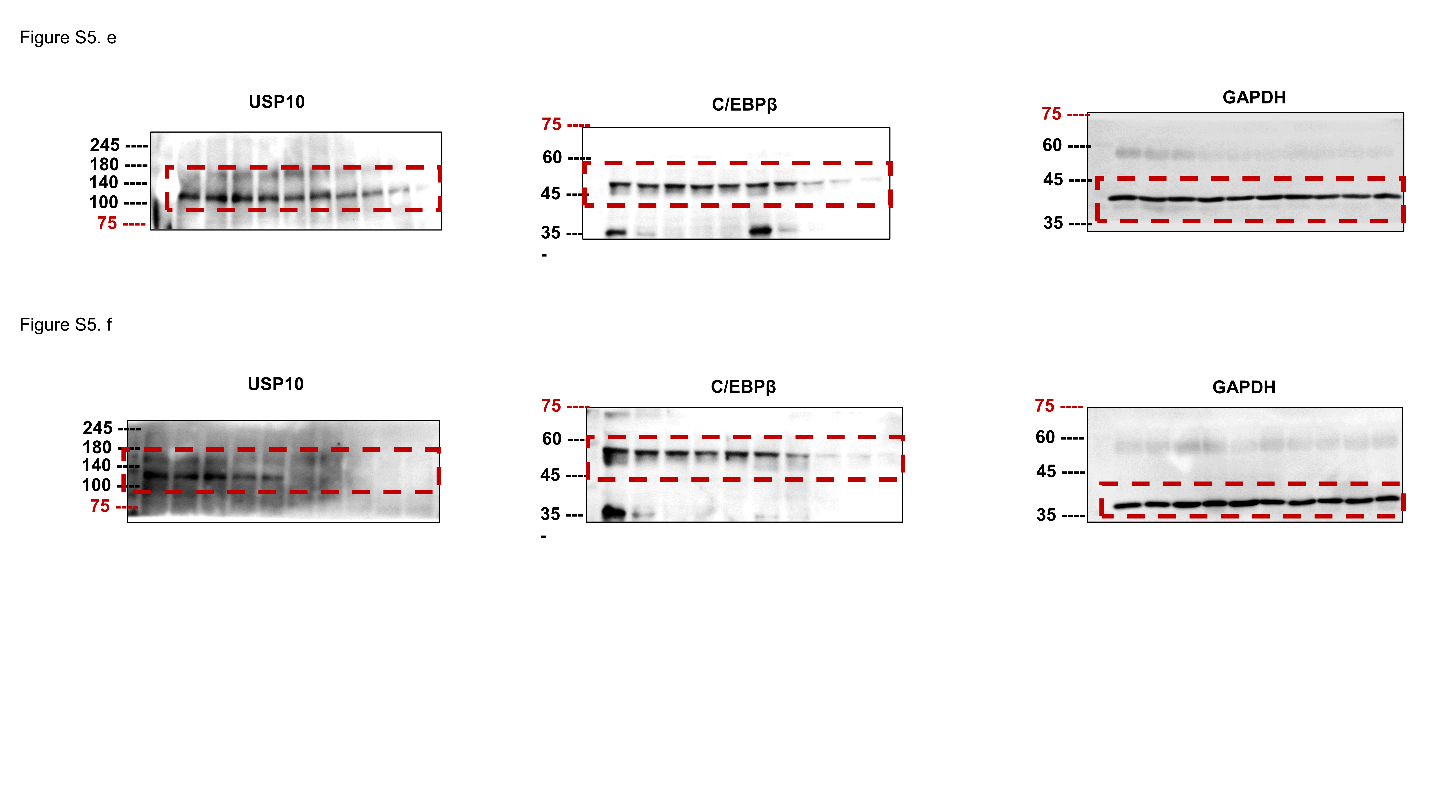


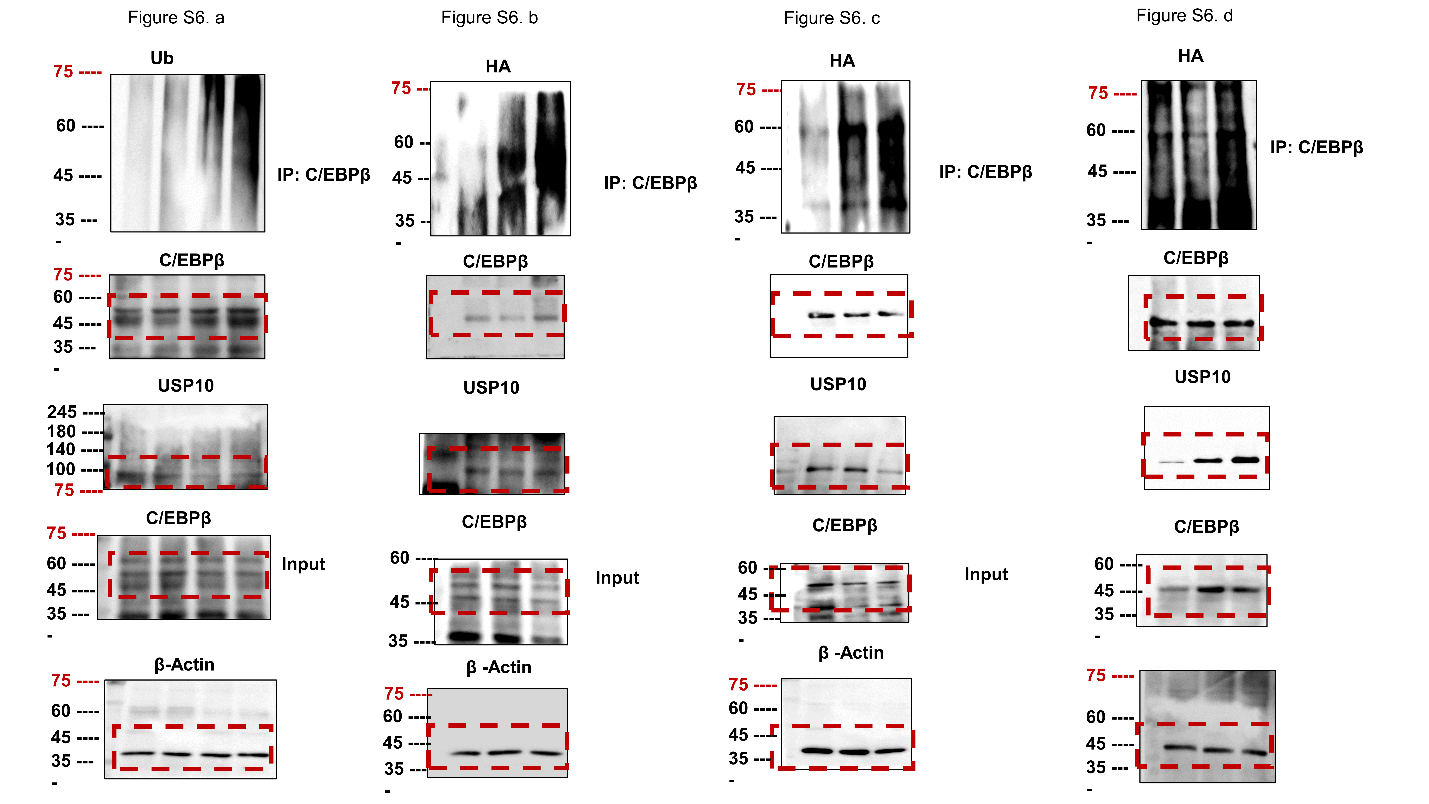


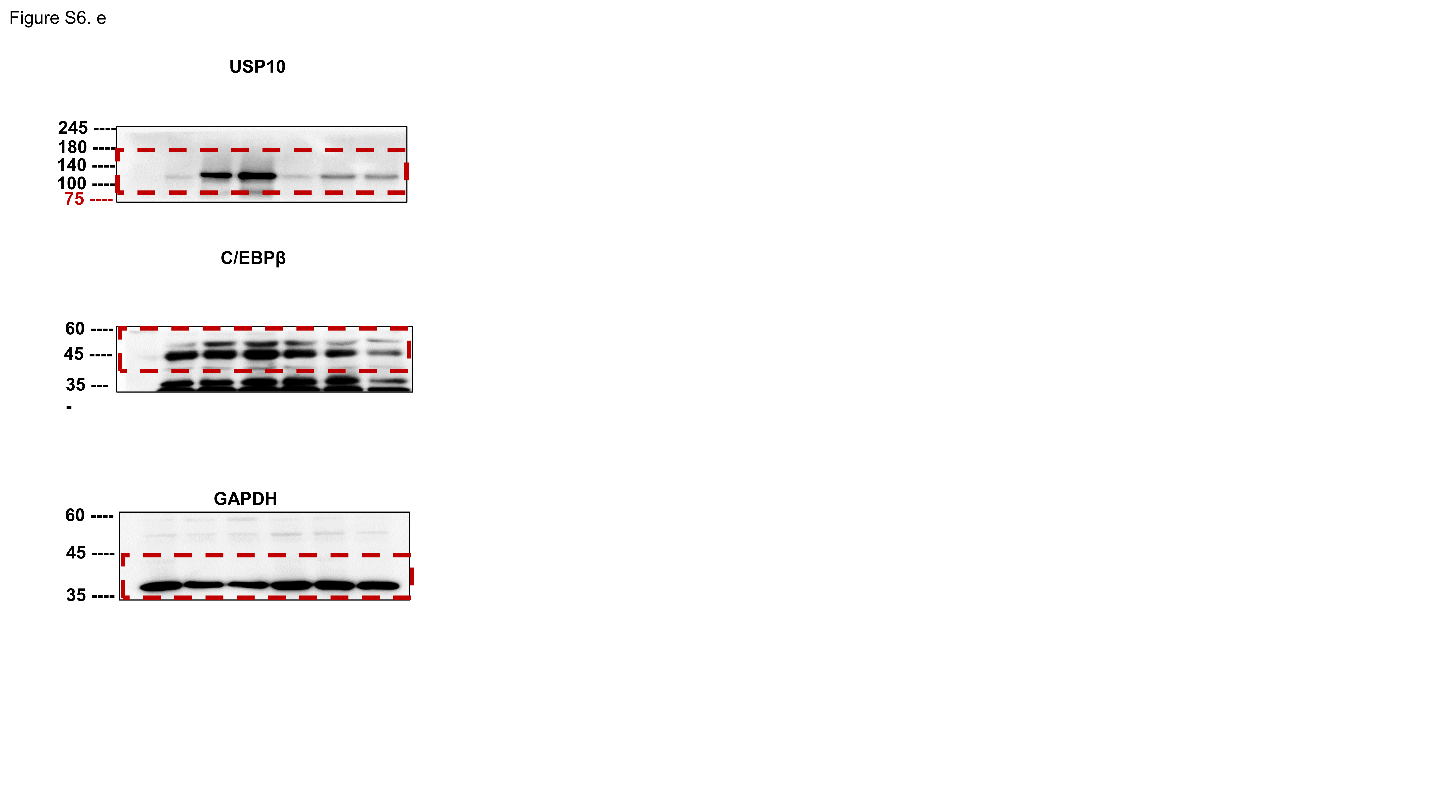

Supplement: Supplementary file 1 — Supplementary Material 1. [file 43556_2025_389_MOESM1_ESM.docx]
